# Validity and reliability of using photography for measuring knee range of motion: a methodological study

**DOI:** 10.1186/1471-2474-12-77

**Published:** 2011-04-18

**Authors:** Justine M Naylor, Victoria Ko, Sam Adie, Clive Gaskin, Richard Walker, Ian A Harris, Rajat Mittal

**Affiliations:** 1 South West Sydney Clinical School, University of New South Wales, Liverpool, NSW, Australia; 2 Department of Orthopaedics, Whitlam Orthopaedic Research Centre, Sydney South West Area Health Service, Liverpool Hospital, Liverpool, NSW, Australia; 3 Cancer Institute, New South Wales, Australia

## Abstract

**Background:**

The clinimetric properties of knee goniometry are essential to appreciate in light of its extensive use in the orthopaedic and rehabilitative communities. Intra-observer reliability is thought to be satisfactory, but the validity and inter-rater reliability of knee goniometry often demonstrate unacceptable levels of variation. This study tests the validity and reliability of measuring knee range of motion using goniometry and photographic records.

**Methods:**

Design: Methodology study assessing the validity and reliability of one method ('Marker Method') which uses a skin marker over the greater trochanter and another method ('Line of Femur Method') which requires estimation of the line of femur. Setting: Radiology and orthopaedic departments of two teaching hospitals. Participants: 31 volunteers (13 arthritic and 18 healthy subjects). Knee range of motion was measured radiographically and photographically using a goniometer. Three assessors were assessed for reliability and validity. Main outcomes: Agreement between methods and within raters was assessed using concordance correlation coefficient (CCCs). Agreement between raters was assessed using intra-class correlation coefficients (ICCs). 95% limits of agreement for the mean difference for all paired comparisons were computed.

**Results:**

Validity (referenced to radiographs): Each method for all 3 raters yielded very high CCCs for flexion (0.975 to 0.988), and moderate to substantial CCCs for extension angles (0.478 to 0.678). The mean differences and 95% limits of agreement were narrower for flexion than they were for extension. Intra-rater reliability: For flexion and extension, very high CCCs were attained for all 3 raters for both methods with slightly greater CCCs seen for flexion (CCCs varied from 0.981 to 0.998). Inter-rater reliability: For both methods, very high ICCs (min to max: 0.891 to 0.995) were obtained for flexion and extension. Slightly higher coefficients were obtained for flexion compared to extension, and with the Marker compared to the Line of Femur Method. For intra- and inter-rater reliability, the mean differences (within 2 degrees) and 95% limits of agreement (within 5 degrees) were generally clinically acceptable for both methods.

**Conclusion:**

Photography potentially offers a superior method of measurement over standard goniometry as visualising the centre of the knee is simplified in a two-dimensional plane and the permanent record provides greater assessor transparency as well as opportunity to confer. The Marker and Line of Femur Methods have moderate to substantial validity, but the inter- and intra-rater repeatability for trained observers are excellent with both methods yielding small mean differences with narrow limits of agreement. The Line of Femur Method offers the added advantage that it does not rely on inter-clinician consistency in identifying the greater trochanter.

## Background

Goniometry for measuring knee range of motion (ROM) is well entrenched in the orthopaedic and rehabilitative communities. It is a measure of some importance as severe restriction in range has ramifications for gait [1-3], function [3-5], and the need for manipulation [6,7]. Further, knee flexion and extension ROMs are incorporated into orthopaedic knee scoring tools to assess disease severity [6], are frequently used to track recovery after various knee surgeries [7-10], and are also used as clinical indicators by which to monitor and benchmark physiotherapy or rehabilitative performance [11]. In light of its extensive use, the clinimetric properties of knee goniometry (namely validity, reliability and responsiveness) are critical to appreciate.

The clinimetric properties of goniometry have been reviewed [12,13]. In general, intra-observer reliability of goniometry has been shown to be acceptable [14-19] and this is usually (though not always [18]) provided the observer is practised in the procedure. However, the validity and inter-rater reliability of knee goniometry has not been consistently shown to be impressive nor acceptable [14-24]. Differences between studies in how the latter clinimetric properties were analysed, the goniometric tools used, and even subject body habitus, are likely contributors to the varying results between studies (Table 1). Whilst most studies evaluating the validity of clinical knee goniometry via comparisons with measures obtained from radiographic images (the 'proxy criterion' or 'best available' estimate) have demonstrated a significant correlation using intra-class or Pearson product moment correlations [14,15,17,21,22], the strength of the relationship has varied considerably. Further, studies which provide a clinically relevant contextual estimate of validity [16,23] indicate that the variation between clinical goniometry and the proxy criterion could be as large as 20 degrees [23]. That the radiographic and the clinical measures inevitably use different landmarks in part explains why variation between the two methods is considerable. Rater error, therefore, is not the only source of variation and thus the level of acceptable variation when assessing validity will necessarily be greater than what would be acceptable for inter-rater variability.

**Table 1:** Overview of validity and inter-rater reliability for knee goniometry

Study	Validity	Inter-rater	Goniometric tool and test position
Lavernia et al [23]	Mean difference between radiologic and goniometric measurement up to 13 degrees with wide (up to 20 degrees) 95% limits of agreement	1-way ANOVA method determined a significant difference between raters for visual and measured range of motion	Standard goniometer; supine.
Lenssen et al [24]		Mean difference (95% limits of agreement) between raters differed by position tested and angle: 1.4 degrees (-16.2 to 19 degrees) (flexion/supine); 2.7 (-6.7 to 12.1) (flexion, sitting); 2.2 (-6.2 to 10.6) (extension, sitting)	Long-arm goniometer; sitting and supine.
Edwards et al [16]	22% of the goniometric measurements were different by 5 degrees or more	Correlation coefficient, 0.91 (type not specified).	Standard goniometer; supine.
Brosseau et al [14]	r = 0.975-0.987 (flexion), r = 0.390-0.514 (extension).	ICC, 0.959-0.982 (flexion); ICC 0.856-0.926 (extension). Both devices performed similarly.	Parallelogram and Universal goniometer; supine.
Brosseau et al [15]	r = 0.73-0.78 (flexion), r = 0.33-0.48 (extension).	ICC, 0.82-0.88 (flexion); ICC 0.43-0.52 (extension). Both devices performed similarly, but the parallelogram provided more advantages to the clinician.	Parallelogram and Universal goniometer; supine.
Watkins et al [19]		ICC 0.90 (flexion); ICC, 0.86 (extension)	Standard goniometer; positioned varied.
Gogia et al [22]	ICC 0.98-0.99.	ICC 0.99	Standard goniometry; side-lying.
Rothstein et al [18]		ICC 0.84-0.93 (flexion); ICC 0.59-0.80 (extension). All devices performed similarly.	3 different standard goniometers; position not specified.
Lawrence, cited by Johnson [17]	r = 0.94 (extension and flexion angles combined).	Intra-observer error +/- 2.3 degrees for end flexion (aggregate standard deviation). Inter-observer error +/- 2.6 degrees end extension and +/-4.2 degrees end flexion	Extended goniometer; supine position.
Cleffken et al [20]		Passive end of range flexion - Smallest detectable difference 0 ± 6.4 degrees	Electronic digital inclinometer; supine

Pearson product moment correlation (r), intra-class correlation (ICC).

Inter-rater goniometric reliability likewise suffers from clinical and statistical uncertainty (Table 1). Inter-rater reliability consistently underperforms intra-rater reliability [14,15,18-20], with inter-rater differences (up to 18 degrees [24]) often exceeding what would be considered a clinically relevant difference. Greater inter-rater reliability for flexion more so than extension angles is typically shown [14,15,18,19,21]. The position of measurement (supine or sitting [24]) and professional background of the assessor [23] have been shown to be confounders. Consequently, the responsiveness of the measure (the ability of the tool to detect a real change) is greatly undermined when different measurers (observers) are involved. In other words, the capacity to detect small changes with standard goniometry when multiple clinicians are involved is highly questionable.

**Figure 1:**
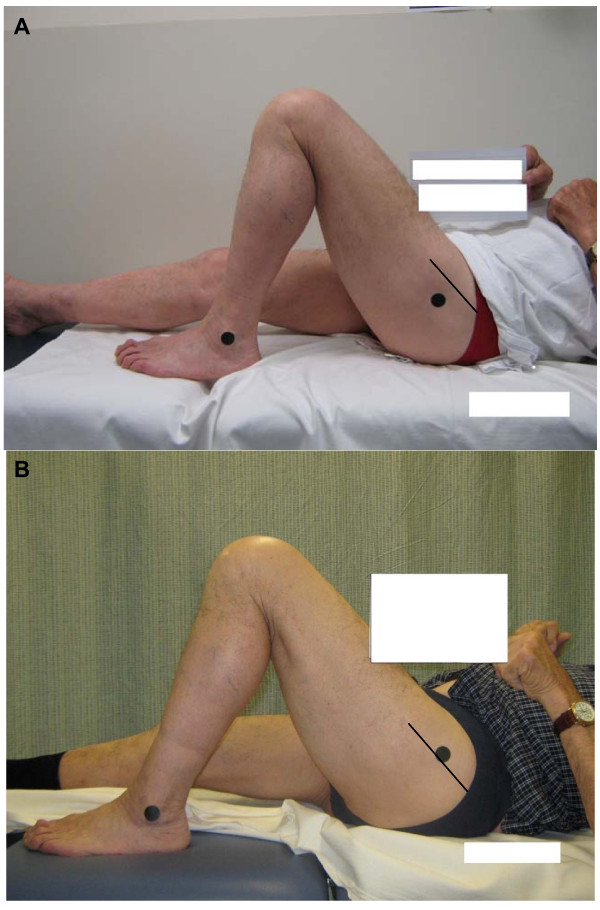
**Inconsistency in GT marker placement between experienced orthopaedic physiotherapists. (Note the position of the marker (black circle) in relation to the scar (black straight line))**.

**Figure 2:**
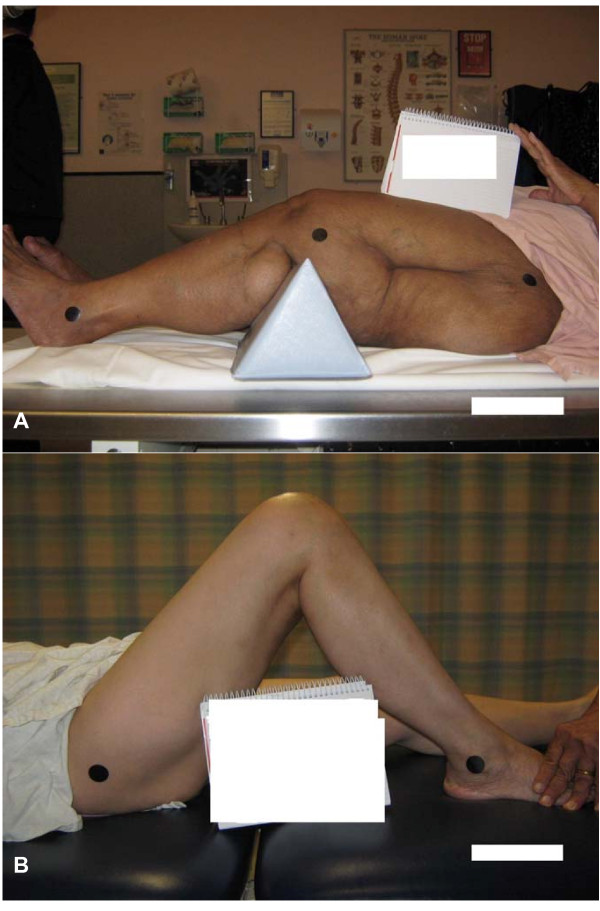
**Different degrees of adiposity influence the ease with which bony landmarks can be identified**.

Agreement between observers for knee goniometry relies largely on consistency in the identification of the bony landmarks on the proximal femur (greater trochanter, GT) and the distal tibia (lateral malleolus, LM), and visualising the sagittal axis of movement for the knee joint. Previous investigators cite the visual estimation of the axis as the 'Achilles heel' of knee goniometry [14,15,24]. Our own anecdotal evidence illuminates the identification of the GT as a primary source of inter-observer error. Figures 1a and 1b illustrate the inconsistency in GT marker placement between experienced orthopaedic physiotherapists. Differing degrees of adiposity overlying the GT often contribute to the difficulty clinicians have in locating the GT in some individuals (Figures 2a and 2b).

In light of the inter-rater reliability concerns in particular, we proposed the use of photographic records for tracking knee ROM. Karkouti and Marks [25] pursued this methodology for measurement of active knee extension and flexion (up to 30 degrees) in standing. They observed fair (ICC 0.40 to 0.59) to very high (ICC 0.8 to 1.0) intra-tester reliability. We propose that the permanent record of knee ROM afforded by a photographic trail provides greater assessor transparency as well as opportunity to confer about measured ROM at a later time if required. We also propose that photography potentially offers a superior method of measurement over standard goniometry as visualising the centre of the knee is simplified in a two-dimensional plane. In this study, we aimed to describe the validity and reliability of two measurement approaches for obtaining knee ROM, both of which used photographic records. One method required raters to measure knee range off a photograph using the skin markers placed over the GT and LM by a clinician as reference points ('Marker Method'). As the identification of GT could itself be a source of error, the second method bypassed the GT marker, requiring the raters to estimate the line of femur instead ('Line of Femur Method'). Both methods required the raters to estimate the centre of the knee joint.

## Methods

### Design and Participants

This methodology study was nested within a large randomised controlled trial investigating the effectiveness of rehabilitation strategies after total knee replacement (TKR) (Australian New Zealand Clinical Trials Registry, ACTRN12609000476235). A subset of patients presenting to the pre-operative education programme prior to their TKR and who consented to participate in the RCT, were invited to partake in this smaller methodology trial. To get a scatter in age and body size, healthy volunteers (onsite healthcare workers) were also invited to participate through word-of-mouth. Pregnancy and inability to speak English were the only exclusion criteria applied. Written informed consent was obtained from all participants and the study was approved as a sub-study within the larger RCT by the Executive of the Human Research Ethics Committee of Sydney South West Area Health Service.

### Protocol

#### Protocol for measuring the participant

On the study day, each participant was positioned (horizontally) supine on a radiographic table in the radiology department. An investigator (JN) arbitrarily positioned the participant's knee (left or right) in either a flexed (between approximately 20-130 degrees) or extended position (between full extension and approximately 20 degrees of flexion). The investigator ensured that the selected position was pain free for the subject, and this position was maintained with use of sandbags and high density foam supports if required. Care was taken using visual inspection to ensure the leg was neither abducted nor adducted, and in neutral rotation. A second investigator [Clinician 1, a physiotherapist of 12 years experience, VK] located and placed adhesive skin markers over the posterior lip of the GT and the middle of the LM. This same investigator took a photograph of the participant's knee whilst in this position using a digital camera (Canon PowerShot A470, 7.1 megapixels, 3.4× optical zoom). The clinician stood between 1 and 1.2 m from the table to get the entire lower limb in full view, and held the camera lens level with and parallel to the participant's knee. The participant maintained the position whilst the markers were removed and a third investigator [Clinician 2, an orthopaedic registrar of 2 years experience, SA] entered the room to repeat the process undertaken by first clinician. Clinician 1 and 2 alternated the sequence for who placed the markers first.

Upon completion of the second photograph, a lateral knee radiograph was taken of the patient in the same position. To minimise the burden on the radiology services, the assessments were spread over two radiology departments utilising similar protocols. The x-ray beam (63 kV, 6.3 mAs) was perpendicular to the plane of the leg and at the level of the knee joint. The radiographs were taken using a 35 × 43 cm plate placed 100 cm from the x-ray beam. The whole body effective dose was <0.02 mSv. Protective covering was provided for the participant.

#### Protocols for measuring the radiographic and photographic angles

**Figure 3:**
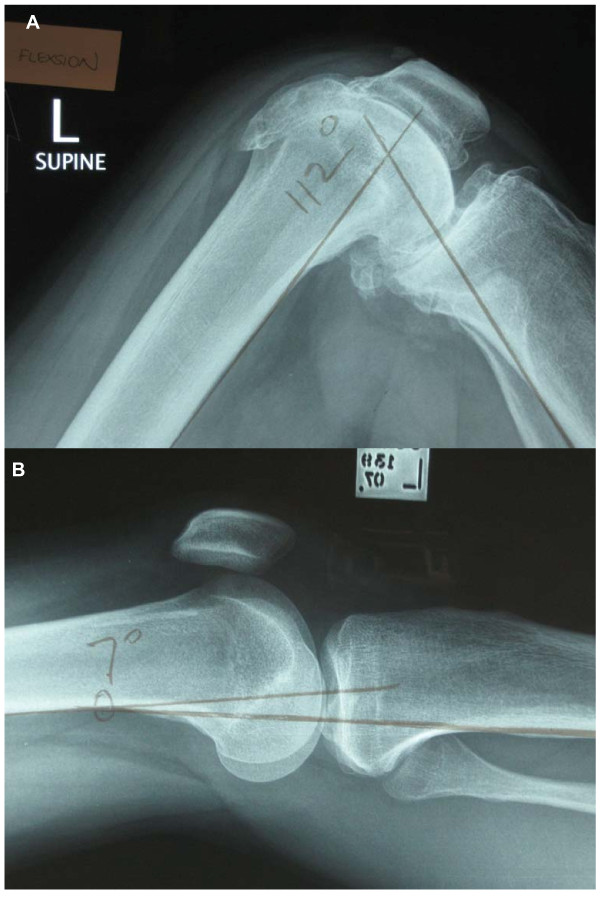
**Patient x-rays demonstrating measurement of angles in flexion and extension**.

A 4^th ^investigator (an orthopaedic surgeon of 15 years, IH), blind to the goniometric measurements, measured the discrete flexion and extension angles off the radiographs using the method utilised by previous investigators [16,23]. In brief, the angle of interest was the angle between the line of the posterior cortex of the femur and the line of the posterior cortex of the tibia (Figures 3a and 3b). Independently, three raters, blinded to each others' measures, obtained knee ROM measurements using a standard goniometer (Whitehall, Model G300) from A5 size photographs printed from a laser printer. To enhance generalisability of the findings, a physiotherapist (VK), an orthopaedic surgeon of 5 years experience (RW), and a research fellow of 7 years experience (JN), made the measurements. For the Marker Method, each rater measured the knee angle by first estimating the axis of the knee on the photograph. For consistency in the interpretation of the axis, it was operationally defined a priori as the 'centre' of the knee on the photograph, approximately midway between the anterior and posterior surfaces, in line with the mid-patella [17]. Each rater then marked the centre on the photograph and then measured the flexion (or extension) angle between the lines drawn from the GT marker to the axis, and the LM marker and the axis. For the Line of Femur Method, the centre of the knee was identified as described above and the rater drew a line between the knee centre and the LM as before. The rater then drew a line from the centre of the knee through what was estimated to be the line of femur. For consistency, this line was operationally defined as originating from the centre of the knee and passing through the long axis of the femur. No further explicit instructions were given as the purpose of this method was to determine if the line of femur could be consistently estimated by different raters in different knees with varying degrees of soft tissue present over the thigh. To check the consistency of each rater for both methods, each rater repeated the measurements on 2 separate occasions (Day 1 and Day 2), a minimum of 1 week apart.

Supplementary to ascertaining the clinimetric properties of measuring angles from photographs, we also took the opportunity to test the consistency between Clinician 1 and Clinician 2 (inter-clinician reliability) in the identification of the bony landmarks. This was done by comparing knee angles measured using the Marker Method from photographs from Clinician 1 with those obtained from Clinician 2. The rater (JN) was kept constant, thus the determination of the centre of the knee was held constant for each patient's pair of measurements. The only sources of any difference, therefore, in the measured angle between clinician photographs were the GT and LM marker placements.

### Sample size, primary outcomes and analyses

The sample size was based on previous studies (n = 10 to 43) evaluating validity or reliability of knee goniometry [16-22,25], thus a minimum sample of 30 heterogeneous participants (varying in age, gender, level of thigh adiposity, and presence of knee arthritis) was considered acceptable. A comparable sample size would permit more meaningful comparison of the estimates of precision between like studies given that sample size - independent of any real differences in performances between goniometric techniques - affects these measures. For the two-way comparisons - validity (radiograph versus individual rater); intra-rater reliability (consistency) (day 1 versus day 2); inter-rater reliability (Clinician 1 versus Clinician 2) - Concordance Correlation Coefficients [26] (CCCs) were used to measure the degree of fit. For the three-way comparisons - inter-rater reliability (3 raters for each of the two methods) - intraclass correlations (ICCs) using the fixed method approach were used [27,28]. Bland and Altman [29] mean difference plus 95% limits of agreement (range of differences of 95% of the paired comparisons) were used to provide clinical estimates of agreement between all paired comparisons. Flexion and extension angles were analysed separately in all comparisons. CCCs or ICCs between 0.60 and 0.80 were considered substantial; coefficients greater than 0.80 were regarded as excellent (near perfect)[30]. A p-value of <0.05 was used to determine statistical significance in mean differences for paired comparisons. No adjustments were made for multiple comparisons as to do so would favour our research hypotheses (in this case, the null hypothesis).

## Results

Thirty-one participants (13 with severe knee osteoarthritis) were recruited. Table 2 summarises the characteristics of the healthy and the arthritic subsets.

**Table 2:** Participant profile

	Arthritic patient volunteers (n = 13)	Healthy volunteers (n = 18)
Age (y)*	64.8 (6.4)	33.8 (7.9)
Body mass index*, kg/m^2^	36.1 (5.4)	23.3 (3.0)
Male, n	7	6

Characteristics of the healthy and the arthritic subsets

* Mean (sd)

Prior to the definitive analyses, the data were checked for normality and statistical outliers. Further, scatter plots were derived to illustrate the closeness between the methods (radiograph or photograph) or the raters. Few cases of non-normal distributions were identified and all paired variables showed a very strong straight line of fit. These details are provided as additional files in the Additional file 1 together with the Bland and Altman (B-A) plots.

The results for the pair-wise comparisons (validity, inter-clinician and intra-rater reliability) are detailed in Table 3. The 95% limits of agreement for all paired comparisons are provided in Table 4.

**Table 3:** Validity

	Mean XRay	Mean Rater	Mean Difference (95% CI)	Diff. p-value (2-sided)	Concordance Correlation Coefficient (95% CI)	B-A Mean Difference (95% Limits of Agreement)
Marker Method						
Flexion (degrees) - XRay > 20						
XRay v RF	90.9	91.5	0.6 (-1.3 to 2.4)	0.54	0.981 (0.970 to 0.993)	1.2 (-7.6 to 10.0)
XRay v PT	90.9	90.1	-0.8 (-3.0 to 1.3)	0.42	0.975 (0.960 to 0.991)	-0.4 (-7.1 to 6.4)
XRay v OS	90.9	90.8	-0.1 (-2.1 to 1.9)	0.93	0.980 (0.966 to 0.993)	1.5 (-6.1 to 9.1)
Extension (degrees) - XRay <= 20						
XRay v RF	6.0	13.8	7.8 (2.2 to 13.5)	0.01	0.540 (0.359 to 0.721)	9 (-6.0 to 24.0)
XRay v PT	6.0	12.1	6.1 (1.2 to 11.0)	0.02	0.611 (0.430 to 0.792)	3 (-9.3 to 15.6)
XRay v OS	6.0	12.8	6.8 (1.9 to 11.7)	0.01	0.586 (0.404 to 0.767)	8 (-9.0 to 25.0)
Line of Femur Method						
Flexion (degrees) - XRay > 20						
XRay v RF	90.9	92.1	1.2 (-0.8 to 3.2)	0.21	0.978 (0.963 to 0.994)	1.0 (-6.0 to 8.1)
XRay v PT	90.9	90.5	-0.4 (-1.9 to 1.2)	0.62	0.988 (0.980 to 0.996)	-0.8 (-10.2 to 8.5)
XRay v OS	90.9	92.4	1.5 (-0.2 to 3.2)	0.08	0.982 (0.971 to 0.994)	-0.1 (-8.9 to 8.7)
Extension (degrees) - XRay <= 20						
XRay v RF	6.0	15.0	9.0 (3.1 to 14.9)	<0.01	0.478 (0.294 to 0.662)	8.9 (-6.7 to 24.6)
XRay v PT	6.0	9.1	3.1 (-1.8 to 8.0)	0.18	0.678 (0.493 to 0.862)	6.1 (-6.5 to 18.7)
XRay v OS	6.0	14.0	8.0 (1.3 to 14.7)	0.02	0.497 (0.305 to 0.689)	6.8 (-5.8 to 19.3)

RF = research fellow; PT = physiotherapist; OS = orthopaedic surgeon; v = versus; B-A = Bland-Altman

**Table 4:** Intra-rater reliability

	Mean Rater Day 1	Mean Rater Day 2	Mean Difference (95% CI)	Difference p-value (2-sided)	Concordance Correlation Coefficient (95% CI)	B-A Mean Difference (95% Limits of Agreement)
Marker Method						
Flexion						
RF Day 1 v Day 2	91.5	91.9	0.5 (-0.1 to 1.1)	0.13	0.997 (0.996 to 1.0)	0.5 (-2.2 to 3.1)
PT Day 1 v Day 2	90.1	90.4	0.3 (-0.2 to 0.8)	0.24	0.998 (0.997 to 1.0)	0.3 (-2.0 to 2.6)
OS Day 1 v Day 2	90.8	89.7	-1.1 (-2.9 to 0.6)	0.19	0.981 (0.967 to 1.0)	-1.1 (-8.9 to 6.6)
Extension						
RF Day 1 v Day 2	13.8	14.9	1.1 (-0.5 to 2.7)	0.14	0.980 (0.966 to 0.995)	1.1 (-2.9 to 5.1)
PT Day 1 v Day 2	12.1	13.3	1.2 (0.0 to 2.4)	0.05	0.983 (0.971 to 0.995)	1.2 (-1.8 to 4.3)
OS Day 1 v Day 2	12.8	14.1	1.3 (-1.6 to 4.2)	0.32	0.941 (0.906 to 0.977)	1.3 (-6.1 to 8.7)
Line of Femur Method						
Flexion						
RF Day 1 v Day 2	92.1	93.0	0.9 (0.0 to 1.7)	0.05	0.995 (0.992 to 0.999)	0.9 (-2.9 to 4.6)
PT Day 1 v Day 2	90.5	90.8	0.3 (-0.3 to 0.9)	0.29	0.998 (0.997 to 1.0)	0.3 (-2.2 to 2.8)
OS Day 1 v Day 2	92.4	92.3	-0.1 (-0.6 to 0.5)	0.73	0.998 (0.997 to 1.0)	-0.1 (-2.5 to 2.3)
Extension						
RF Day 1 v Day 2	15.0	16.4	1.4 (0.2 to 2.7)	0.03	0.983 (0.971 to 0.995)	1.4 (-1.7 to 4.6)
PT Day 1 v Day 2	9.1	9.3	0.2 (-0.9 to 1.2)	0.72	0.990 (0.983 to 0.997)	0.7 (-2.5 to 2.9)
OS Day 1 v Day 2	14.0	14.4	0.4 (-1.1 to 2.0)	0.54	0.987 (0.978 to 0.996)	0.4 (-3.6 to 4.5)

RF = research fellow; PT = physiotherapist; OS = orthopaedic surgeon; v = versus; B-A = Bland-Altman

### Validity

#### Radiographic versus Photographic measurements

For flexion, pair-wise comparisons between the radiographic measurements and both photographic methods for all 3 raters yielded near-perfect CCCs, varying from 0.975 to 0.988. The mean differences between all the paired comparisons were small and not statistically different, varying from 0.09 to 1.5 degrees. For the B-A computations, the mean difference was within 1.6 degrees on all occasions and the 95% limits of agreement were within 5 to 10 degrees.

For extension, the pair-wise comparisons yielded moderate to substantial CCCs, varying from 0.478 to 0.678. Both methods and all raters performed similarly. The mean differences were larger than those observed for flexion, varying from 3.1 to 9.0 degrees with most comparisons achieving statistical significance. For the B-A computations, the mean difference was within 10 degrees on all occasions and the 95% limits of agreement were wider than those observed for flexion, varying from 6 to 25 degrees.

### Reliability

#### Intra-rater reliability for photographic measurements (Day 1 vs Day 2)

For flexion and extension angles, near-perfect CCCs were attained for all 3 raters for both methods with slightly greater CCCs seen for flexion (CCCs varied from 0.981 to 0.998). Mean day-to-day differences approximated zero, and on all almost all occasions (11/12 comparisons) were not significantly different. For the B-A computations, the mean difference was within 1.5 degrees on all occasions and the 95% limits of agreement were within 5 degrees in 10 of 12 comparisons.

#### Inter-rater reliability for photographic measurements

The results of the 3-way comparisons are detailed in Table 5. For both methods, near-perfect ICCs (min to max: 0.891 to 0.995) were obtained for flexion and extension angles. Slightly higher coefficients were obtained for flexion compared to extension, and with the Marker Method compared to the Line of Femur Method. The B-A computations for inter-rater limits of agreement yielded narrow limits for flexion and extension for the Marker Method (flexion - mean difference within 1.5 degrees (95% limit of agreement within 5 degrees); extension - mean difference within 1.8 degrees (95% limit of agreement within 6 degrees), and slightly broader limits for the Line of Femur Method (flexion - mean difference within 2 degrees (95% limit of agreement within 7.3 degrees); extension - mean difference within 6 degrees (95% limit of agreement within 12.9 degrees)).

**Table 5:** Inter-rater reliability (3 raters): Marker Method v Line of Femur Method

	Mp	Mr	ICC
Marker Method			
Flexion	1310.1	2.36	0.995
Extension	351.3	2.03	0.983
Line of Femur Method			
Flexion	1376.6	3.53	0.992
Extension	370.9	14.48	0.891

Mp, mean square between patients; Mr, mean square of residuals; ICC, intra-class correlation

#### Inter-clinician reliability for placement of markers

For flexion and extension angles, near-perfect CCCs were obtained (flexion, 0.986; extension, 0.962), indicating very high consistency in marker placement. The mean differences were not significantly different (Clinician 1, 91.4 v Clinician 2, 90.2 degrees flexion, p = 0.51; Clinician 1, 13.9, v Clinician 2, 14.7 degrees extension, p = 0.72). Narrow limits of agreement were obtained - mean difference -1.2 (-7.6 to 5.3) degrees for flexion; mean difference 0.72 (-5.1 to 6.6) degrees for extension.

## Discussion

The measurement of knee ROM by multiple observers for tracking outcomes after TKR necessitates the use of a robust method of measurement. Not only should the method demonstrate reasonable validity, but the method must be reliable in different hands. Here, we set out to determine the validity and reliability of knee ROM measurements using photographic records as we proposed that this approach may yield superior clinimetric properties to the more typical goniometric approaches reported in the literature.

Our results indicate that measuring ROM off a photograph, regardless of whether the GT landmark is used, has excellent validity for flexion as indicated by the CCCs and the B-A computations. Validity for extension angles is less impressive. That our limits of agreement appear superior to that reported by Lavernia et al [23] is partly explained by the fact that we compared discrete flexion and extension angles, whereas the former compared total ROM which itself will produce a level of error which is potentially a summation of the errors associated with both flexion and extension measurements. That the limits of agreement were not narrower than was observed is also worthy of comment. Had we been able to obtain a single radiograph that was the length of the lower limb, the method used to measure the knee angle from the radiograph would have been a better approximation of the method used to measure angles off the photographs. As neither radiology department possessed a radiographic plate of sufficient size, the radiographic measurement could only utilise the distal third of the posterior border of the femur and the proximal posterior border of the tibia. In this light, the radiographic comparison is predisposed to produce a difference. What becomes important then is what method, if any, produces a smaller difference. This criticism is likely to apply to previous studies utilising radiographic comparisons capturing the knee only. That all raters performed similarly in the validity domain is perhaps the most important finding here as it is between-rater consistency which is of most concern for comparing change across time.

The intra-rater reliability for either method was excellent, again consistent with most of the previous research [14-20]. Likewise, inter-rater reliability for either method was excellent, both yielding similar or superior inter-rater reliability outcomes reported by several others [15,16,18-20]. High inter-rater reliability for the Marker Method at least should be expected to some extent given that for this method, the only clinically important source of inter-rater error will be the estimation of the axis of the knee. This partly explains why both our study and the studies by Brosseau and colleagues [14,15] yield similarly high coefficients. Brosseau et al [14,15] had markers placed over bony landmarks by one independent observer and the same markers were used by all raters. However, in our study, even when the GT marker was not used, inter-rater reliability was very high. It would appear then, that inter-rater reliability is assisted by the use of photographic records. Visual inspection of the rater photographic records indicates that the raters (despite differences in professional leanings) were typically close in their approximation of the knee centre with only slight variation evident for the line of femur estimation. This in part likely reflects compliance with the measuring rules applied, but also suggests parallax error due to measuring a 3-dimensional figure on a 2-dimensional landscape is small provided the photograph is taken under standardised conditions.

Like others, we found inter-rater reliability and the radiographic comparisons better for flexion angles. The reasons why flexion angles seem less susceptible to observer variation are partly mathematical and partly speculative. On one level, correlation coefficients are affected by the range of the measuring scale [26], with larger ranges associated with higher correlation coefficients. As the range of extension angles is small (here 20°, measured between 0 and 20 degrees), the coefficients for extension compared to flexion measurements are inevitably smaller. On another level, we also contend that visualising the line of femur and the centre of the knee is simpler in flexion; the overlying thigh soft tissue would seem to approximate the line of femur more closely, and the knee joint can be viewed as a circle, making the estimation of the centre easier.

We note that Enwemeka [21] attributed poorer performance in validity outcomes for extension compared to flexion angles to the accompanying knee rotation which itself changes the relative position of the relevant bony landmarks. The radiological comparisons undertaken in the latter study were made in standing, and yet the skin markers were placed when the subjects were supine. Consequently, a difference between goniometric measurements made in standing (based on markers placed in supine) and radiological measurements made in standing were inevitable. The same considerations do not apply for our study. This notwithstanding, the GT does move in relation to the overlying skin when the knee moves from extension to flexion in supine. It is imperative then, that the GT marker (if used) is re-positioned between extension and flexion measurements in the same individual. This adjustment was not necessary here as only one flexion or extension angle was obtained in each individual subject.

Though the primary focus here was to test the clinimetric properties of measuring ROM from a photograph, we took the opportunity to assess the inter-clinician consistency of marker placement. Interestingly, the level of agreement between the two clinicians was very high, unlike our previous experience in the clinic. That the clinicians were consistent with their placement of the markers in this study is probably explained by the fact that both clinicians were highly familiar with the process, and were under overt and high-level scrutiny (that is, were under laboratory conditions) and thus were highly focused on their approach. The therapists in our previous example located the markers in the course of their usual work-day stressors and environment, and were perhaps less aware that their method for identifying bony landmarks (most notably, the GT) may be suboptimal.

## Conclusions

In terms of practice recommendations, both approaches manifested impressive clinimetric properties. In addition, the photographic approach provides a permanent and transparent recovery trail providing the opportunity for clinicians or researchers to confer where doubt exists either for GT location or the line of femur estimate. We therefore contend that these features combined render both photographic methods potentially superior to the alternative goniometric methods currently in use. We recommend that if only one clinician or several well trained clinicians are identifying the GT, the Marker Method is preferable owing to its slightly better clinimetric performance overall (Tables 5 and 6). The inter-rater differences were not statistically significantly different and 95% of scores were within 5 degrees for both flexion and extension for the Marker Method. The line of Femur Method behaved similarly for flexion but produced larger mean differences (up to 5 degrees) and larger limits of agreement (up to 13 degrees) for extension. The intra- and inter-rater reliability for marker placement would need to be established first, however, if the Marker Method is utilised. As the Line of Femur approach does not require real-time identification of the GT, we recommend the use of this method when knee ROM measurements are to be taken by multiple assessors of varying expertise. In our experience, it is easier to train and test the reliability of one person in the method of measuring off a photograph using the Line of Femur Method than it is to adequately train many assessors in the clinic to correctly identify bony landmarks as is necessary for the Marker Method. It must be noted that neither photographic method exonerates the clinician or observer at the time of assessment from a standardised approach. Patient positioning, photographic technique and care in obtaining the limits of extension and flexion (whether they are passively or actively obtained) remain important.

**Table 6:** Inter-rater limits of agreement

	B-A Mean Difference (95% Limit of Agreement)	B-A Mean Difference (95% Limit of Agreement)
Marker Method	Flexion	Extension
RF v PT	1.4 (-2.0 to 4.8)	1.7 (-1.8 to 5.2)
RF v OS	0.7 (-4.0 to 5.3)	1.1 (-2.8 to 4.9)
PT v OS	-0.8 (-4.2 to 2.7)	-0.7 (-3.1 to 1.7)
Line of Femur Method		
RF v PT	1.6 (-4.0 to 7.2)	5.9 (0.3 to 11.5)
RF v OS	-0.3 (-4.3 to 3.7)	1.0 (-4.0 to 6.0)
PT v OS	-1.9 (-5.6 to 1.8)	-4.9 (-12.8 to 3.0)

RF = research fellow; PT = physiotherapist; OS = orthopaedic surgeon; v = versus;

B-A = Bland-Altman

Finally, in a recent randomised trial evaluating the effect of prosthesis design on ROM after TKR, Chaudary et al [31] justified their study largely on the basis that the method of measurement of knee ROM used in previous trials and the error associated with the measurement, may contribute to the conflicting results concerning knee ROM and prosthesis type. We echo these criticisms and urge investigators to adopt a standardised, simple and transparent approach such as the methods described herein as this would do much to advance our understanding of the links between ROM and prosthesis design, surgical approach or even rehabilitative interventions.

## Competing interests

The authors declare that they have no competing interests.

## Authors' contributions

JMN contributed to the design, collection of data, the analysis and interpretation of data. VK, SA and RW contributed to the design, data collection and interpretation. CG contributed to the data analysis. IAH contributed to data collection. RM contributed to the interpretation of data and prepared the draft for publication. All authors contributed to manuscript preparation. All authors read and approved the final manuscript.

## Pre-publication history

The pre-publication history for this paper can be accessed here:

http://www.biomedcentral.com/1471-2474/12/77/prepub

## Supplementary Material

Additional file 1 **Bland and Altman (B-A) and scatter plots**. The Bland and Altman (B-A) plots were derived to illustrate the closeness between the methods (radiograph or photograph) or the raters. Few cases of non-normal distributions were identified and all paired variables showed a very strong straight line of fit. Click here for file
