# Supplementary material for: Validity and reliability of using photography for measuring knee range of motion: a methodological study
Source: BMC Musculoskelet Disord. 2011 Apr 18;12:77. doi: 10.1186/1471-2474-12-77 (PMC3095577; doi:10.1186/1471-2474-12-77)
Supplement: Additional file 1 — Bland and Altman (B-A) and scatter plots. The Bland and Altman (B-A) plots were derived to illustrate the closeness between the methods (radiograph or photograph) or the raters. Few cases of non-normal distributions were identified and all paired variables showed a very strong straight line of fit. [file 1471-2474-12-77-S1.PDF]

Legend for all
B-A = Bland and Altman
RF = research fellow
PT = physiotherapist
OS = orthopaedic surgeon
D = day
Fem = line of femur method
Marker = marker method
All axes are degrees

Knee Measurement Study: Bland-Altman Plots, Extension Measurements.

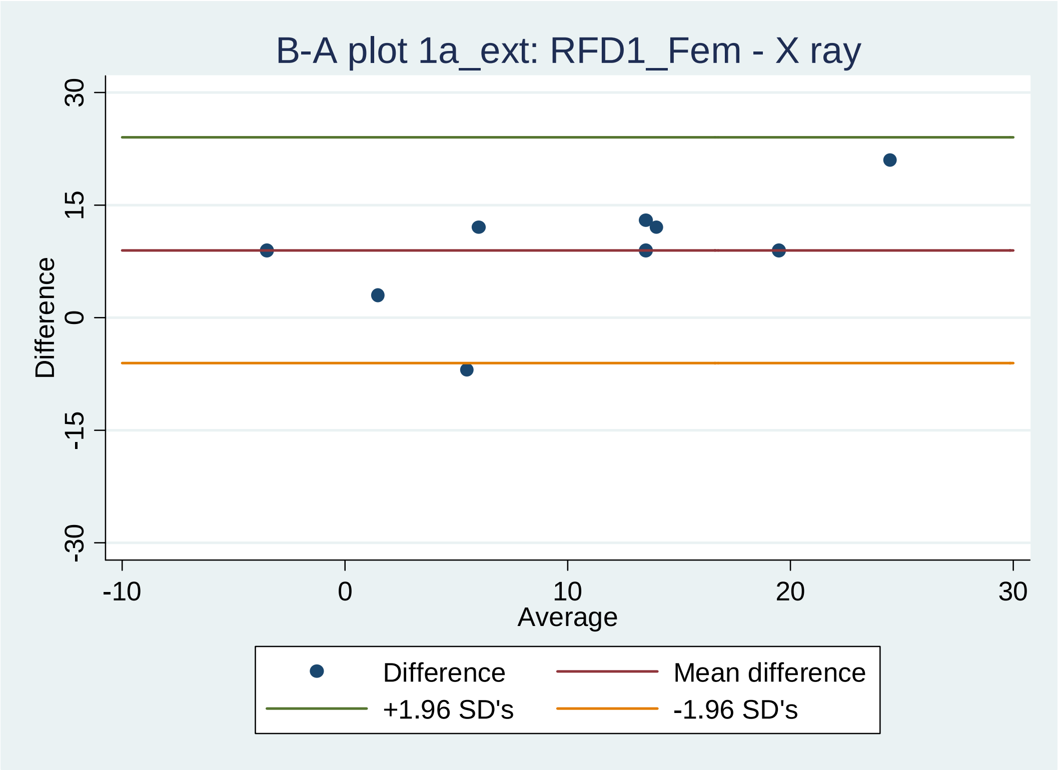

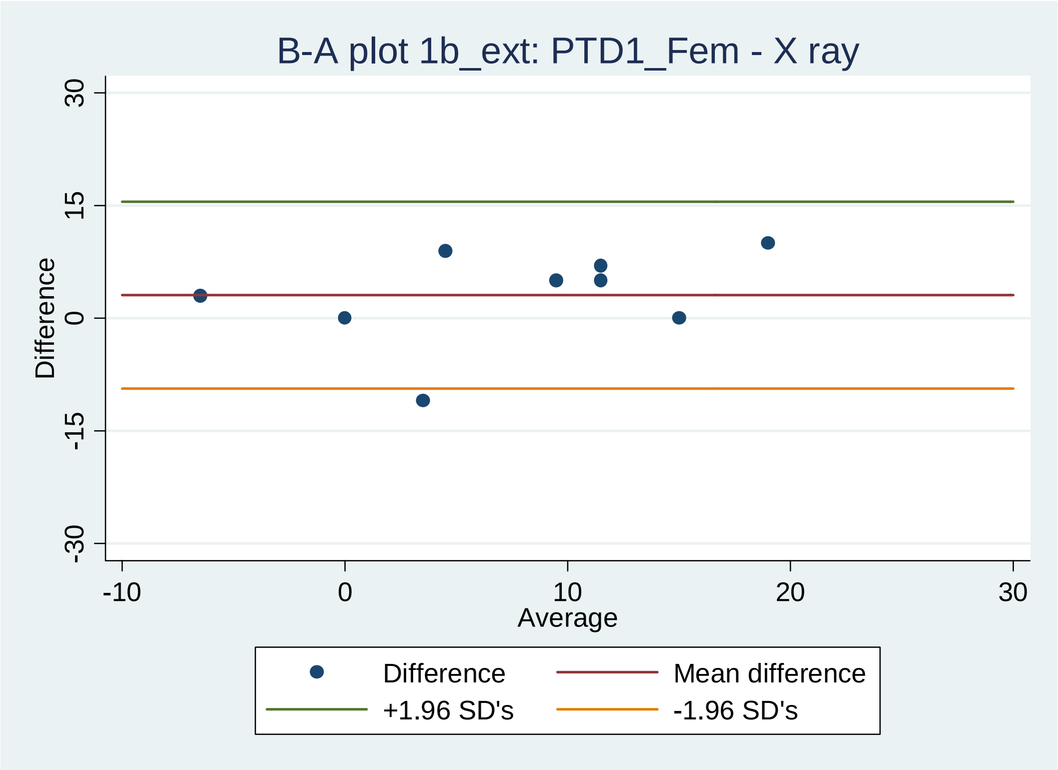

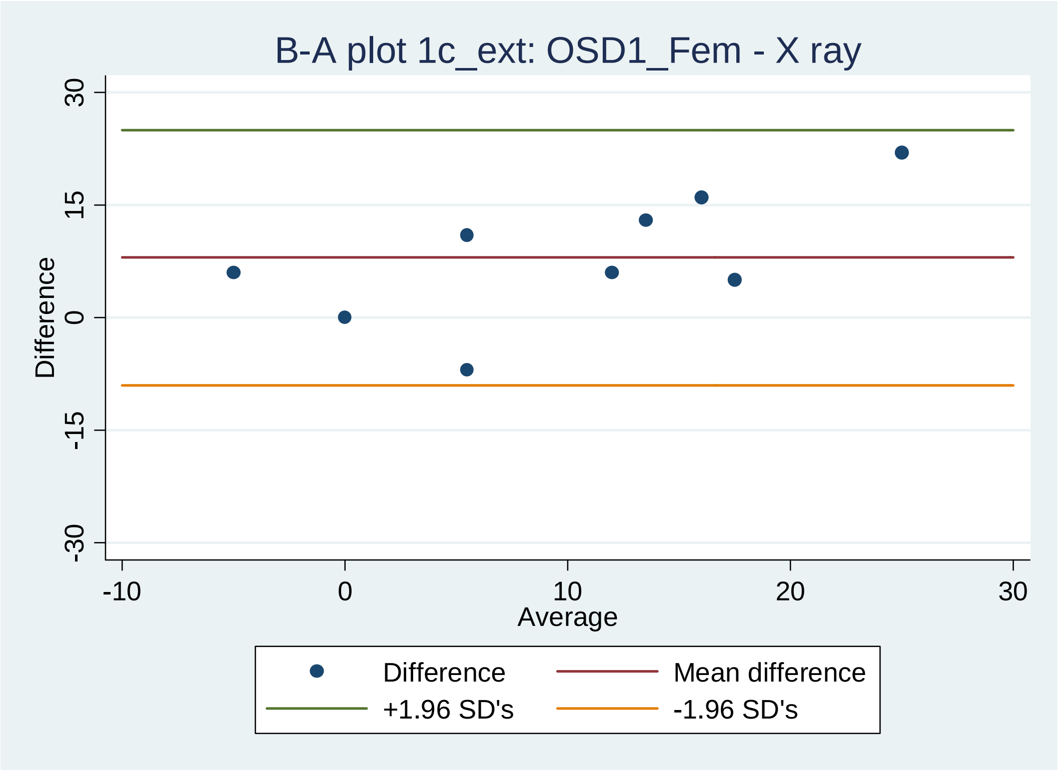
B-A plot 1c_ext: OSD1_Fem - X ray
Difference
30
15
0
-15
-30
-10
0
10
20
30
Average
Difference
Mean difference
+1.96 SD's
-1.96 SD's

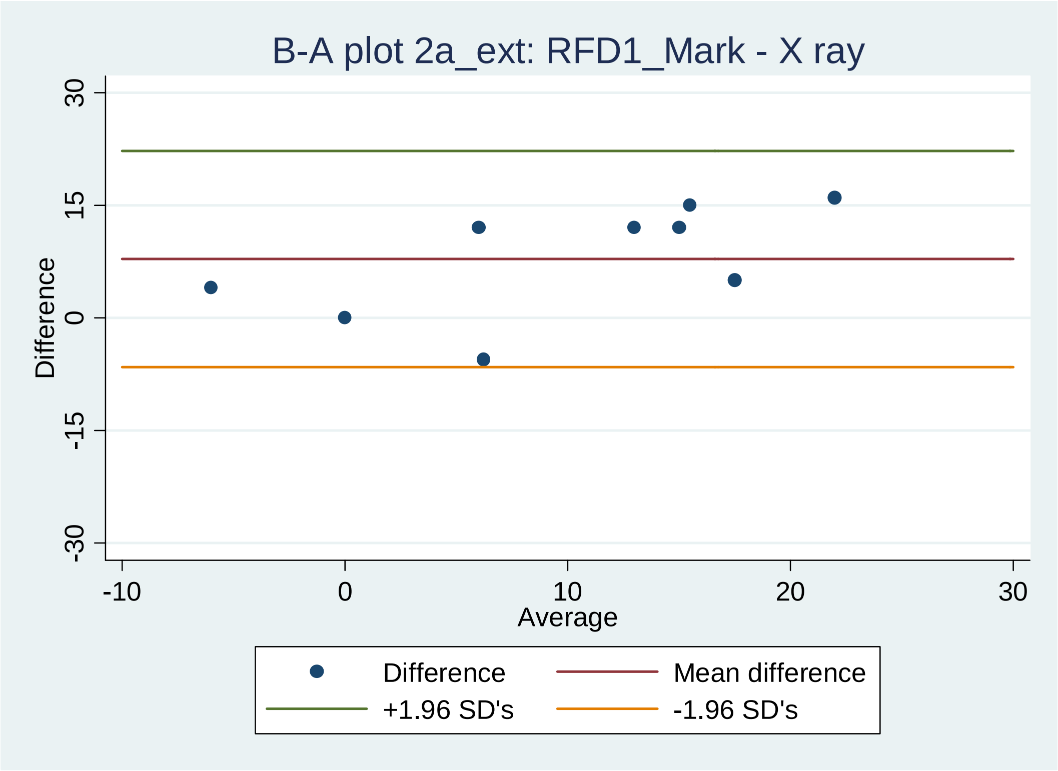
B-A plot 2a_ext: RFD1_Mark - X ray
Difference
30
15
0
-15
-30
-10
0
10
20
30
Average
Difference
Mean difference
+1.96 SD's
-1.96 SD's

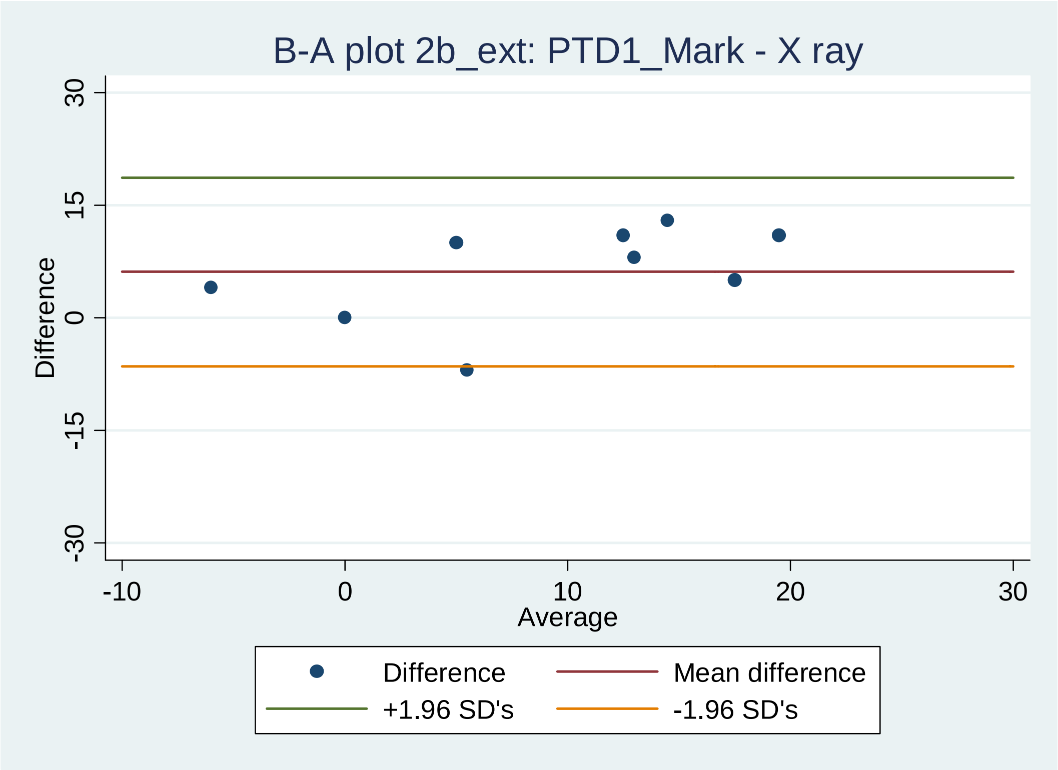
B-A plot 2b_ext: PTD1_Mark - X ray
Difference
30
15
0
-15
-30
-10
0
10
20
30
Average
Difference
Mean difference
+1.96 SD's
-1.96 SD's

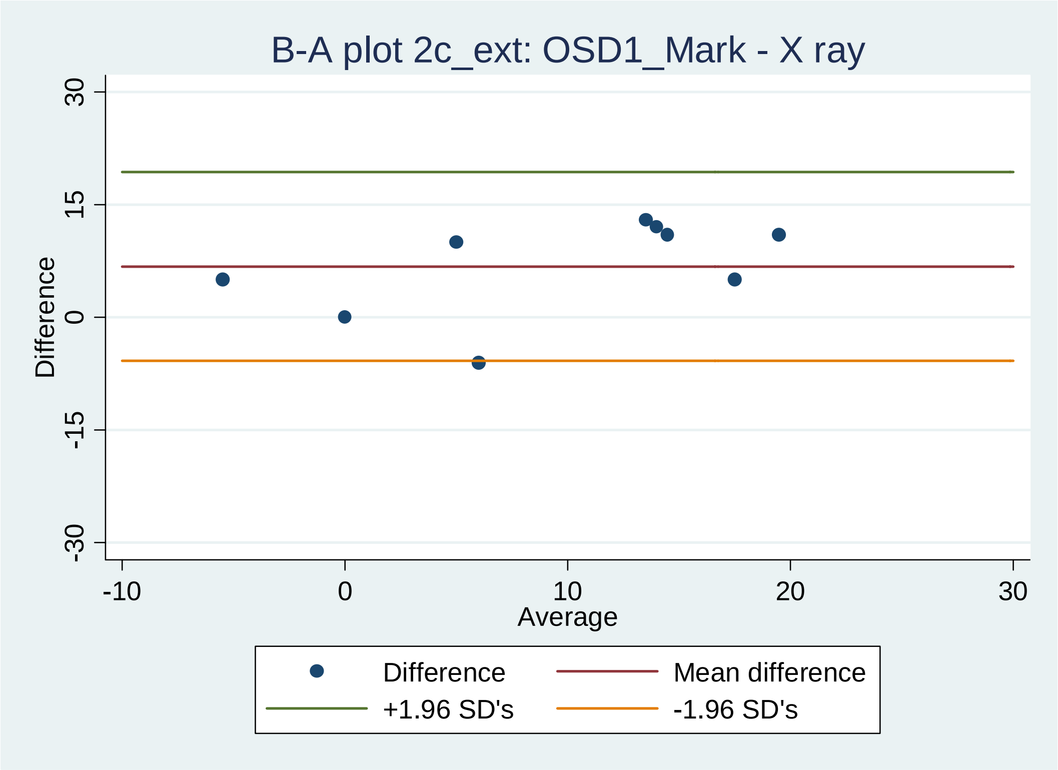
B-A plot 2c_ext: OSD1_Mark - X ray
Difference
30
15
0
-15
-30
-10
0
10
20
30
Average
Difference
Mean difference
+1.96 SD's
-1.96 SD's

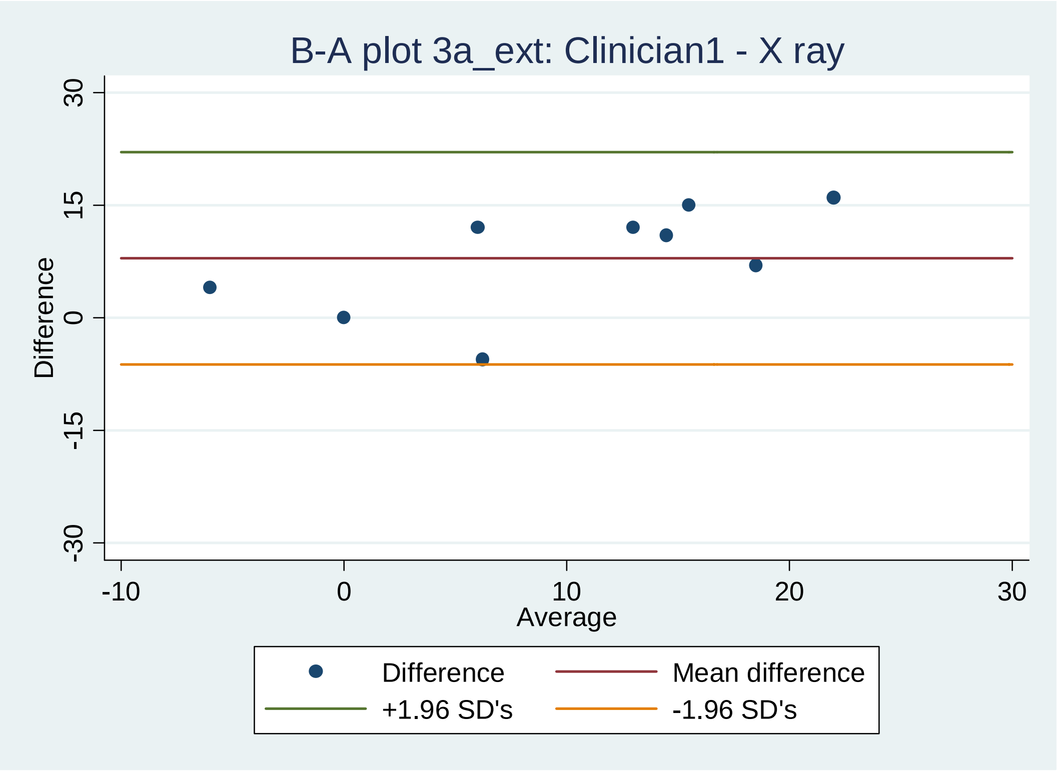
B-A plot 3a_ext: Clinician1 - X ray
30
15
0
-15
-30
Difference
-10
0
10
20
30
Average
Difference
Mean difference
+1.96 SD's
-1.96 SD's

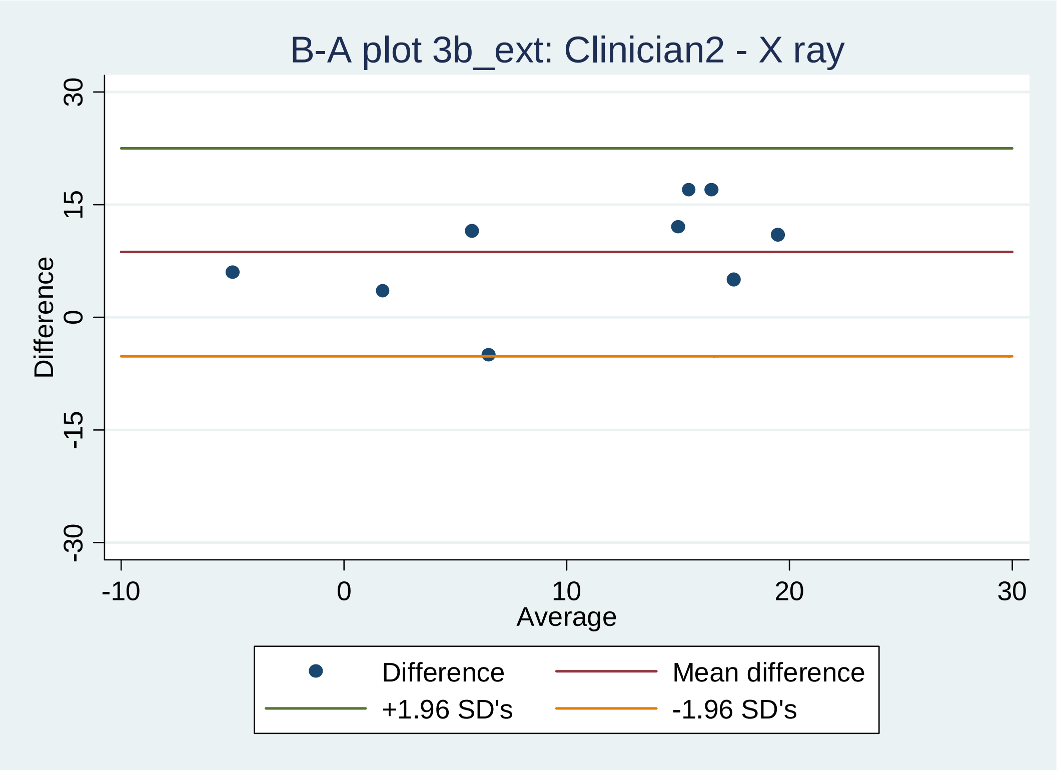
B-A plot 3b_ext: Clinician2 - X ray
30
15
0
-15
-30
Difference
-10
0
10
20
30
Average
Difference
Mean difference
+1.96 SD's
-1.96 SD's

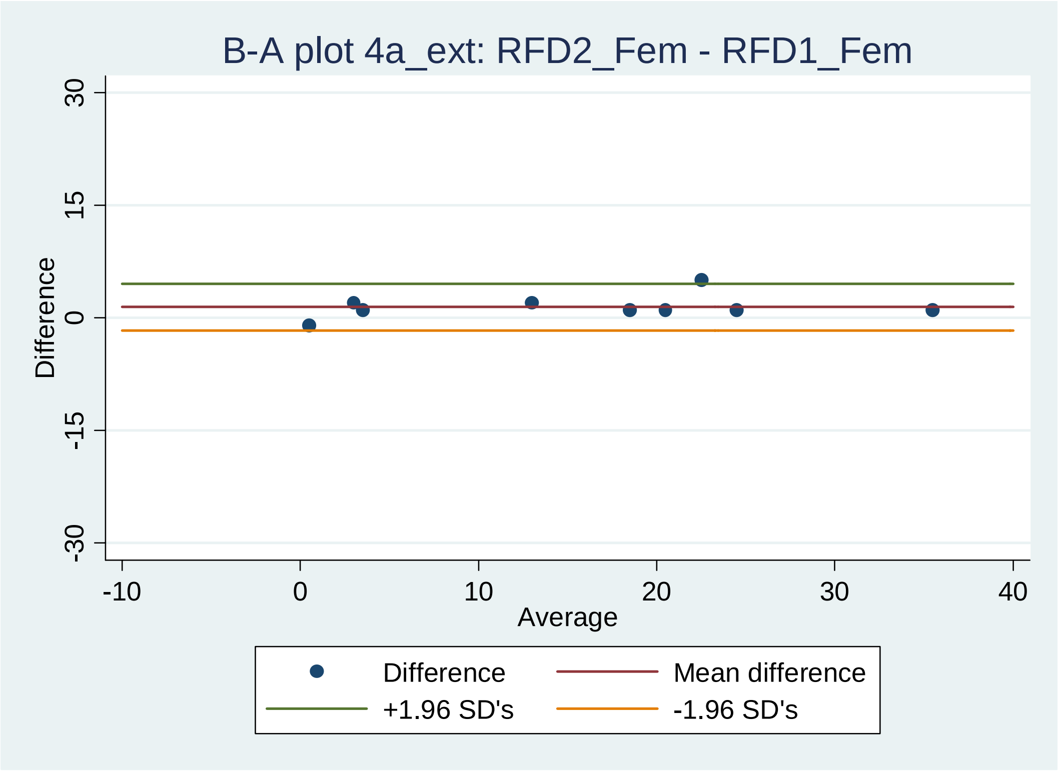
B-A plot 4a_ext: RFD2_Fem - RFD1_Fem
Difference
30
15
0
-15
-30
-10
0
10
20
30
40
Average
Difference
Mean difference
+1.96 SD's
-1.96 SD's

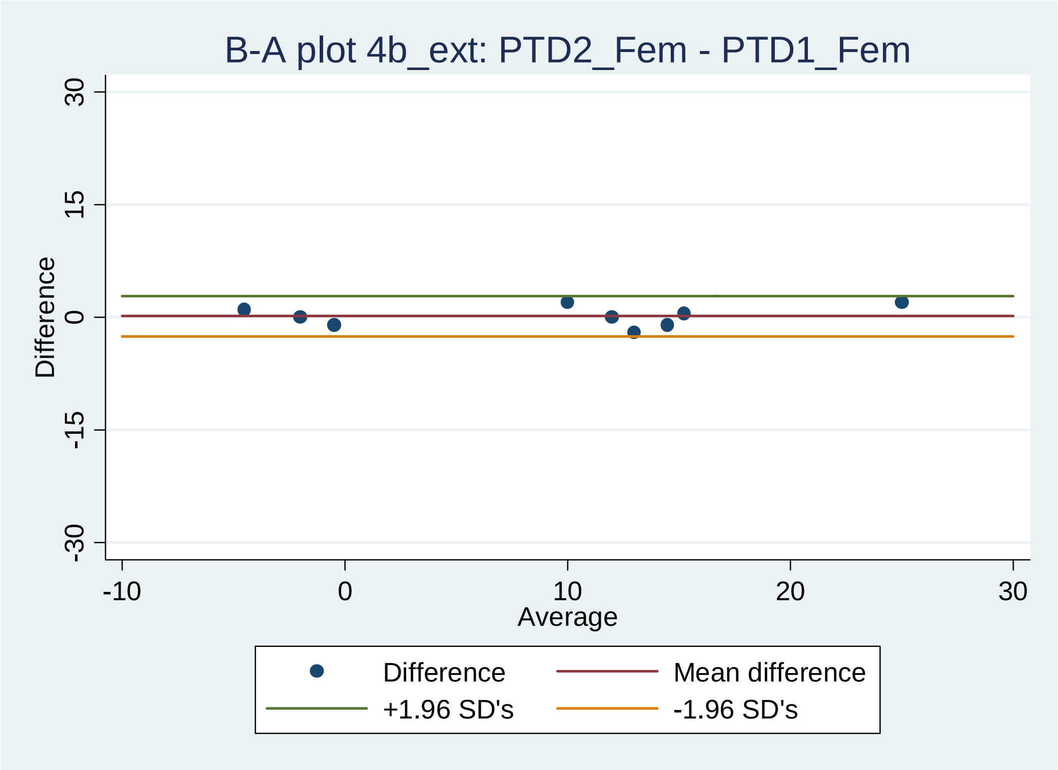
B-A plot 4b_ext: PTD2_Fem - PTD1_Fem
Difference
30
15
0
-15
-30
-10
0
10
20
30
Average
Difference
Mean difference
+1.96 SD's
-1.96 SD's

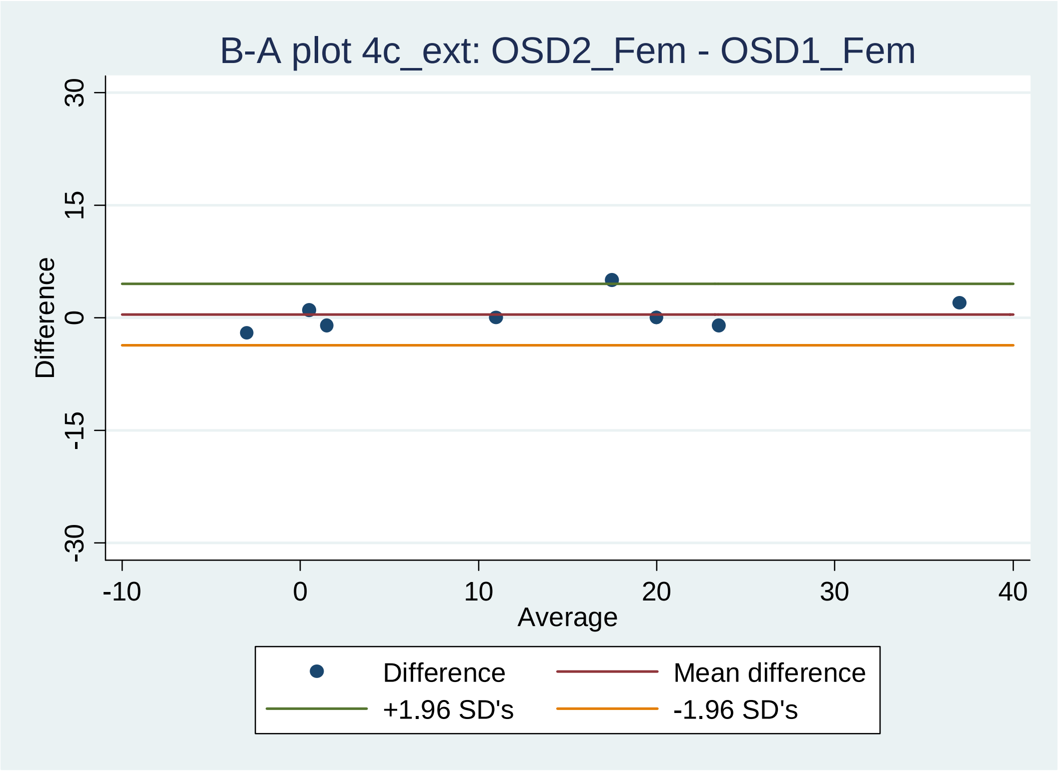
B-A plot 4c_ext: OSD2_Fem - OSD1_Fem
Difference
30
15
0
-15
-30
-10
0
10
20
30
40
Average
Difference
Mean difference
+1.96 SD's
-1.96 SD's

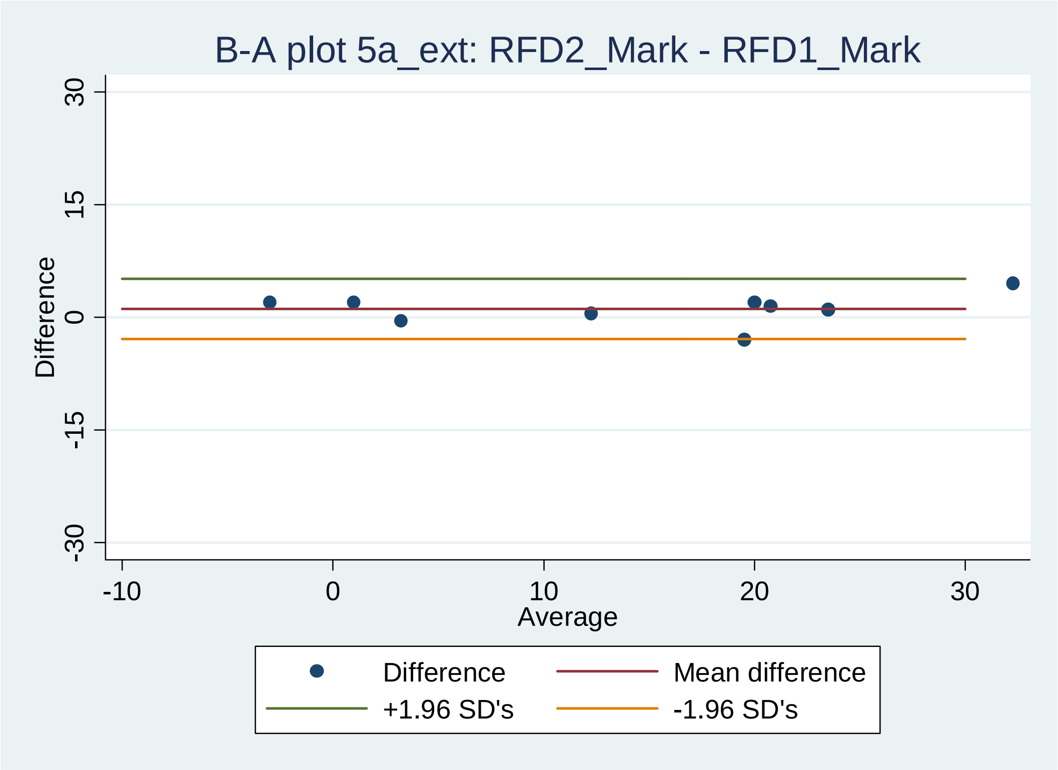
B-A plot 5a_ext: RFD2_Mark - RFD1_Mark
Difference
30
15
0
-15
-30
-10
0
10
20
30
Average
Difference
Mean difference
+1.96 SD's
-1.96 SD's

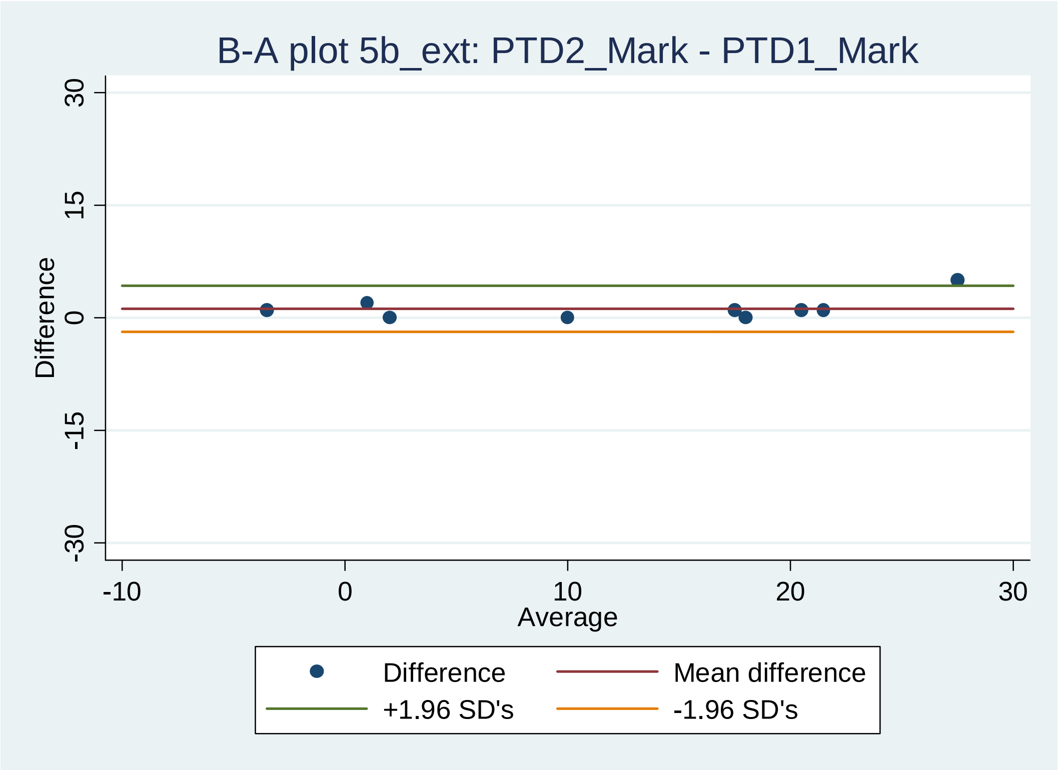
B-A plot 5b_ext: PTD2_Mark - PTD1_Mark
Difference
30
15
0
-15
-30
-10
0
10
20
30
Average
Difference
Mean difference
+1.96 SD's
-1.96 SD's

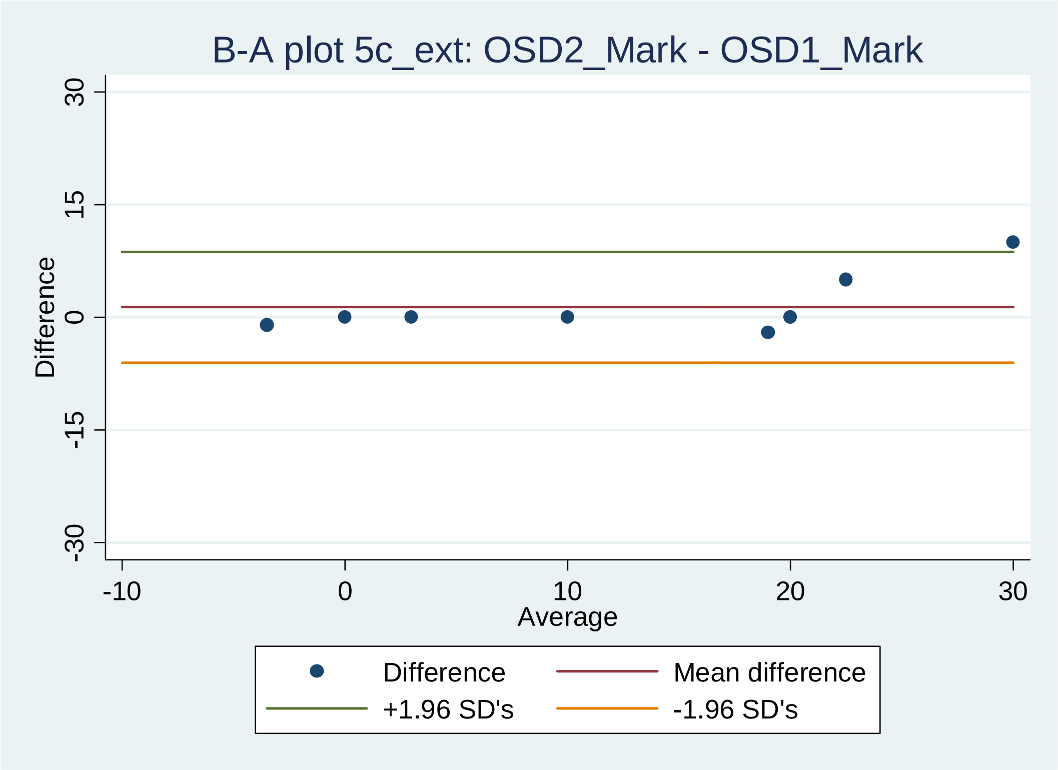
B-A plot 5c_ext: OSD2_Mark - OSD1_Mark
Difference
30
15
0
-15
-30
-10
0
10
20
30
Average
Difference
Mean difference
+1.96 SD's
-1.96 SD's

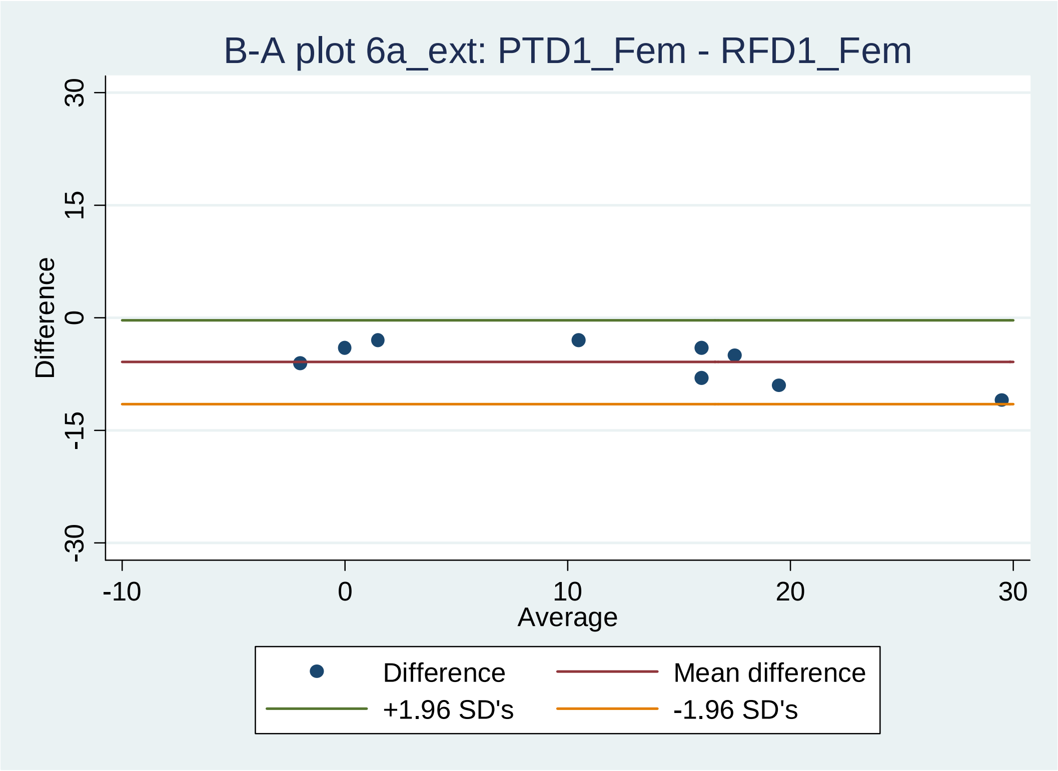
B-A plot 6a_ext: PTD1_Fem - RFD1_Fem
Difference
30
15
0
-15
-30
-10
0
10
20
30
Average
Difference
Mean difference
+1.96 SD's
-1.96 SD's

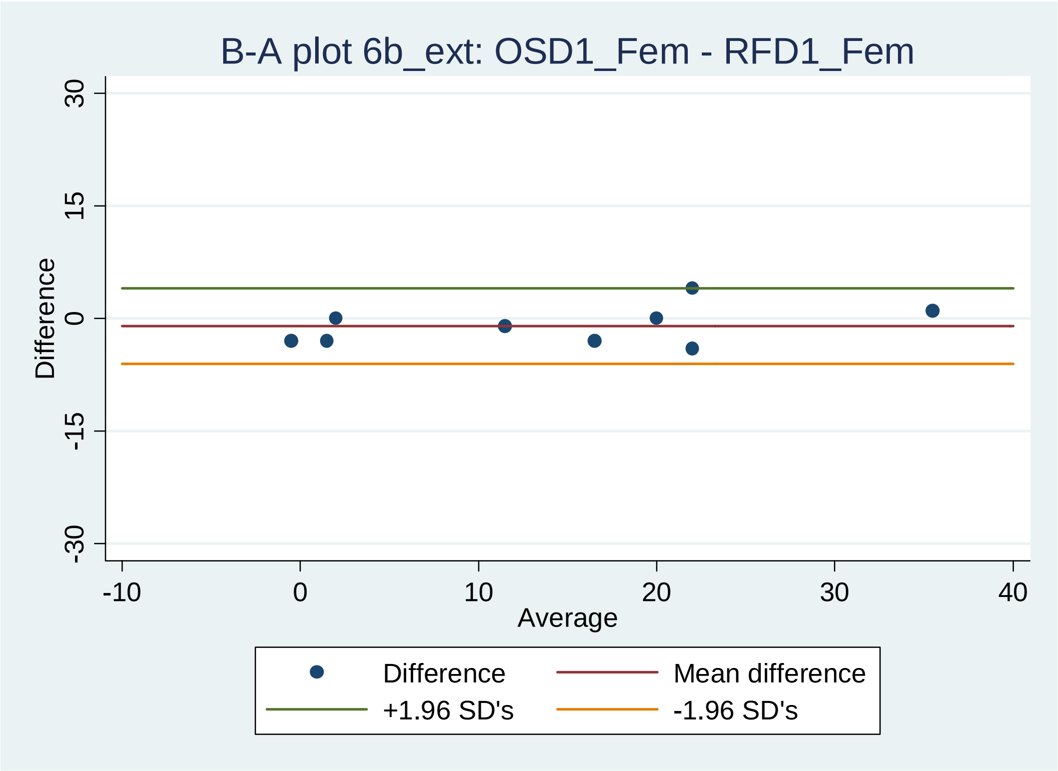
B-A plot 6b_ext: OSD1_Fem - RFD1_Fem
Difference
30
15
0
-15
-30
-10
0
10
20
30
40
Average
Difference
Mean difference
+1.96 SD's
-1.96 SD's

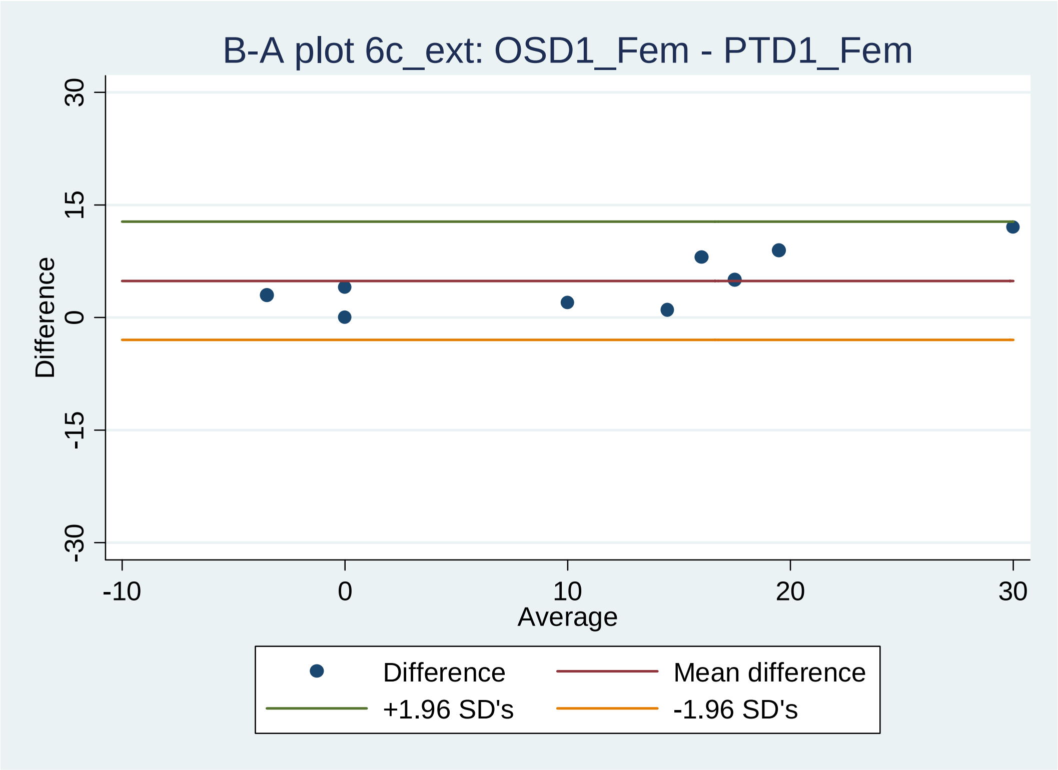
B-A plot 6c_ext: OSD1_Fem - PTD1_Fem
Difference
30
15
0
-15
-30
-10
0
10
20
30
Average
Difference
Mean difference
+1.96 SD's
-1.96 SD's

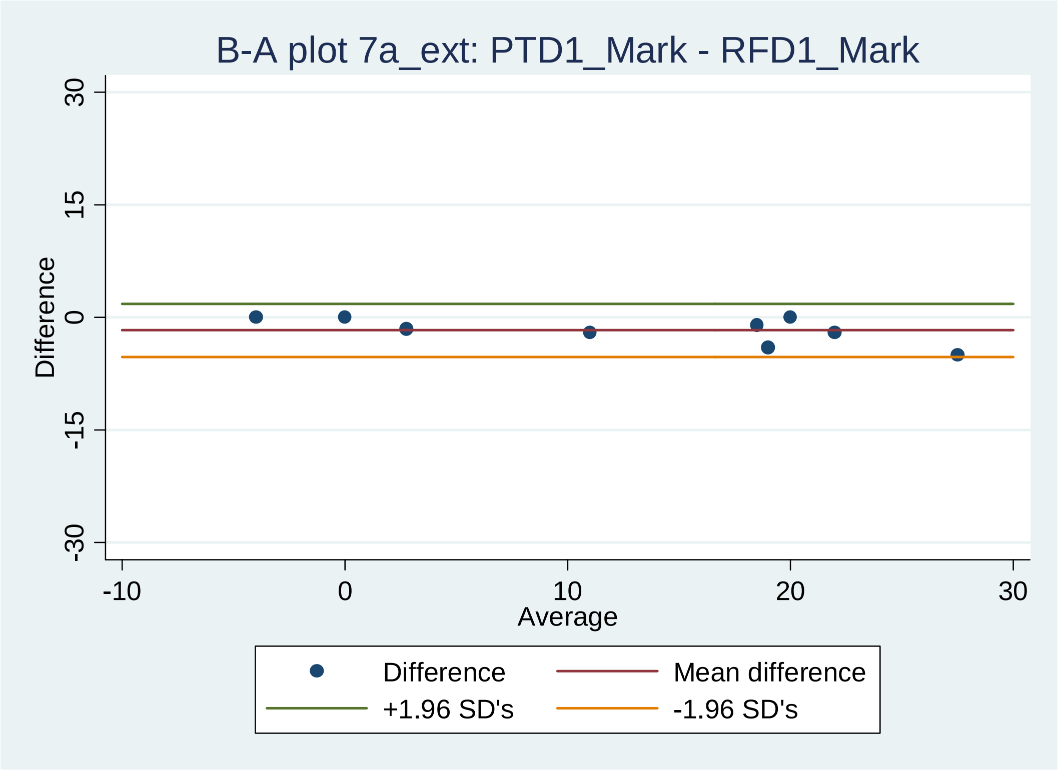
B-A plot 7a_ext: PTD1_Mark - RFD1_Mark
Difference
30
15
0
-15
-30
-10
0
10
20
30
Average
Difference
Mean difference
+1.96 SD's
-1.96 SD's

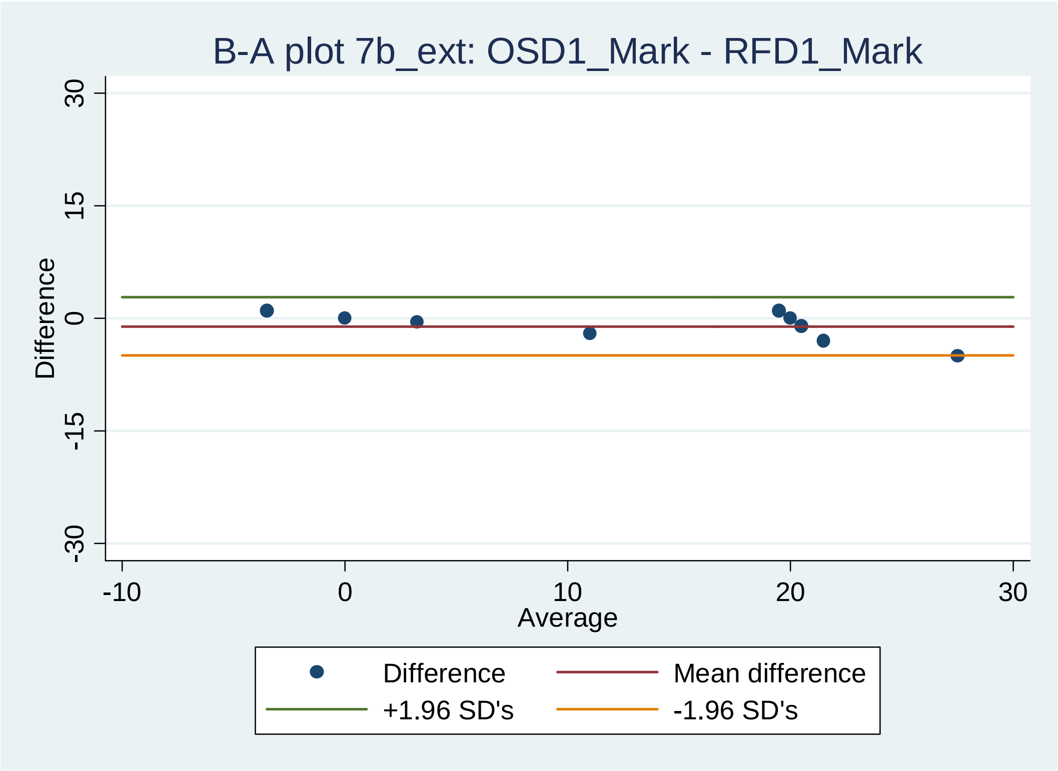
B-A plot 7b_ext: OSD1_Mark - RFD1_Mark
Difference
30
15
0
-15
-30
-10
0
10
20
30
Average
Difference
Mean difference
+1.96 SD's
-1.96 SD's

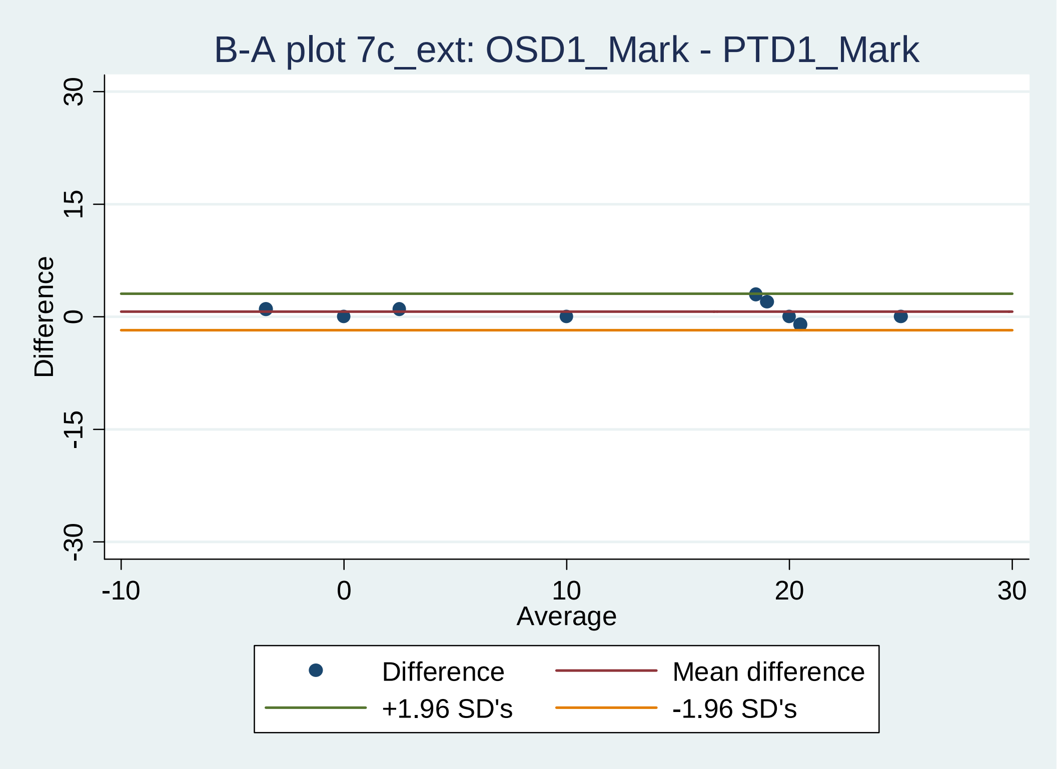
B-A plot 7c_ext: OSD1_Mark - PTD1_Mark
Difference
30
15
0
-15
-30
-10
0
10
20
30
Average
Difference
Mean difference
+1.96 SD's
-1.96 SD's

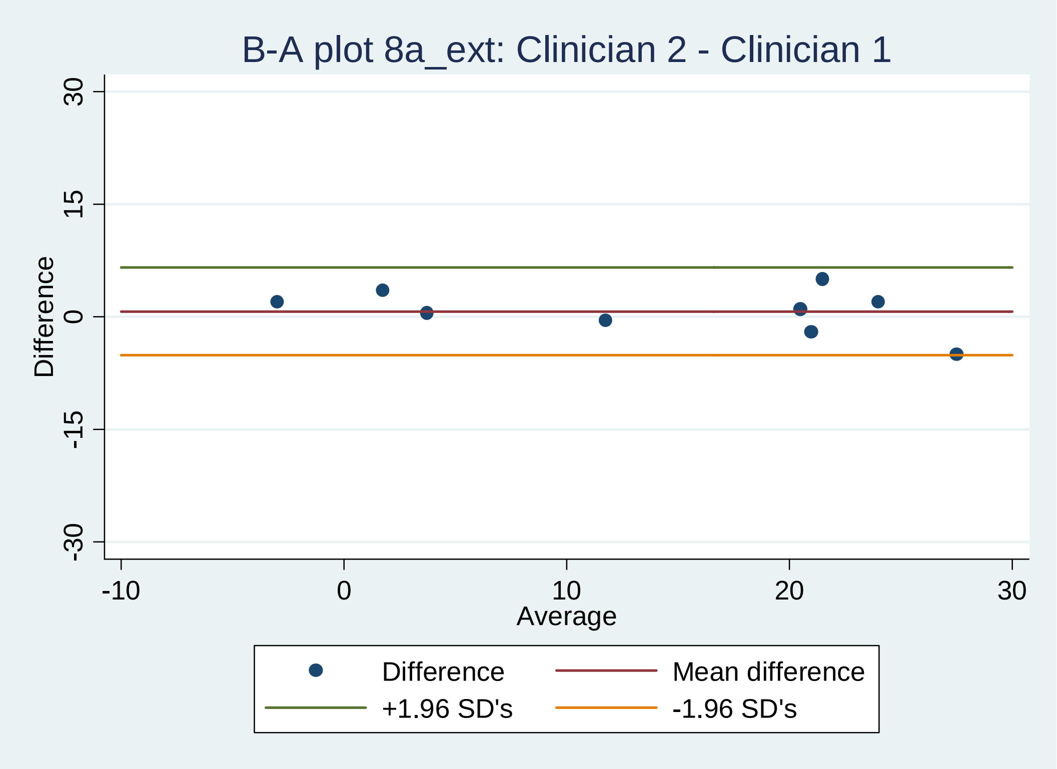
B-A plot 8a_ext: Clinician 2 - Clinician 1
Difference
30
15
0
-15
-30
-10
0
10
20
30
Average
Difference
Mean difference
+1.96 SD's
-1.96 SD's

Knee Measurement Study: Bland-Altman Plots, Flexion Measurements.

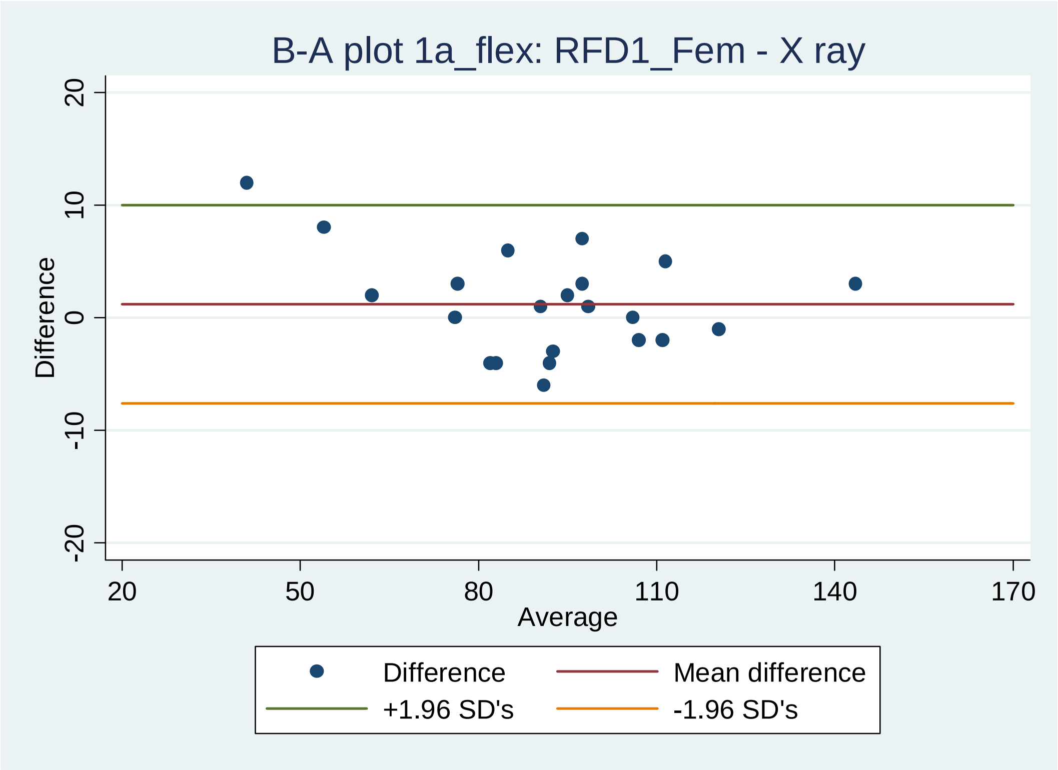

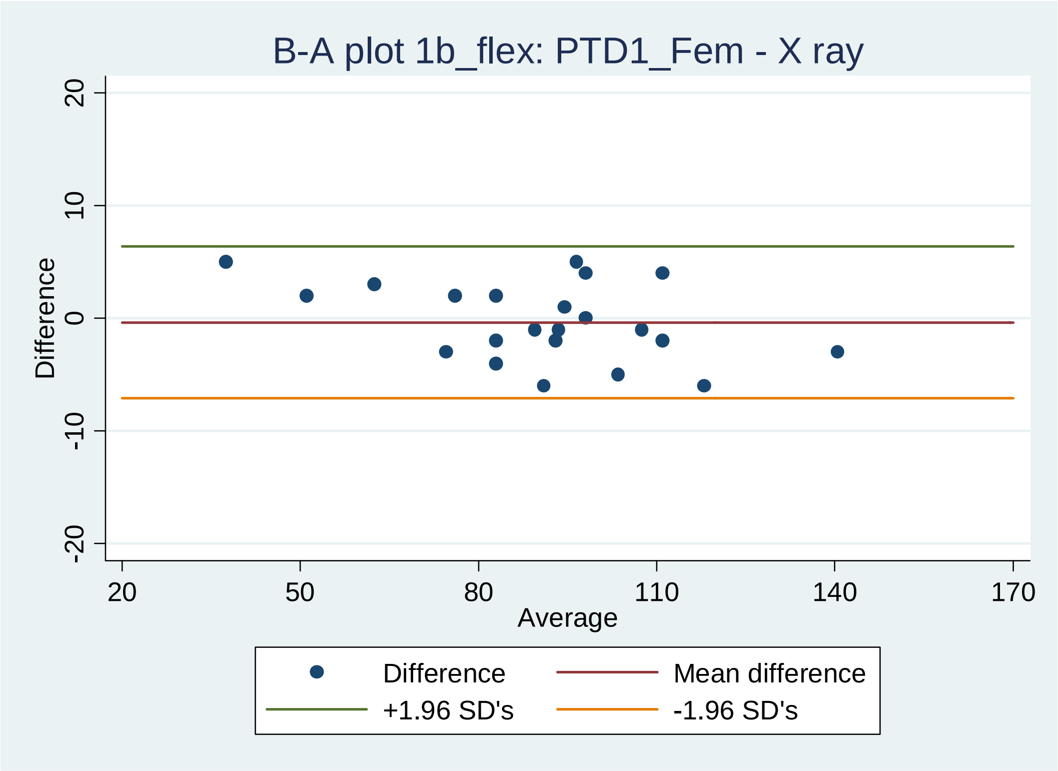

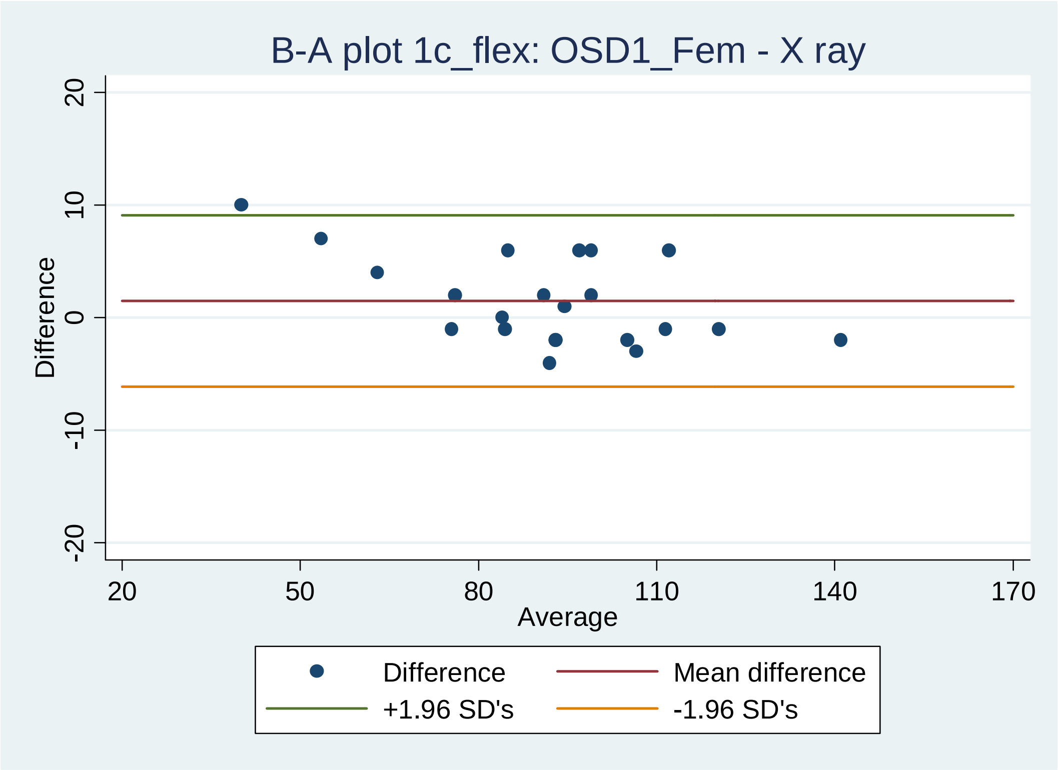
B-A plot 1c_flex: OSD1_Fem - X ray
Difference
20
10
0
-10
-20
20
50
80
110
140
170
Average
Difference
Mean difference
+1.96 SD's
-1.96 SD's

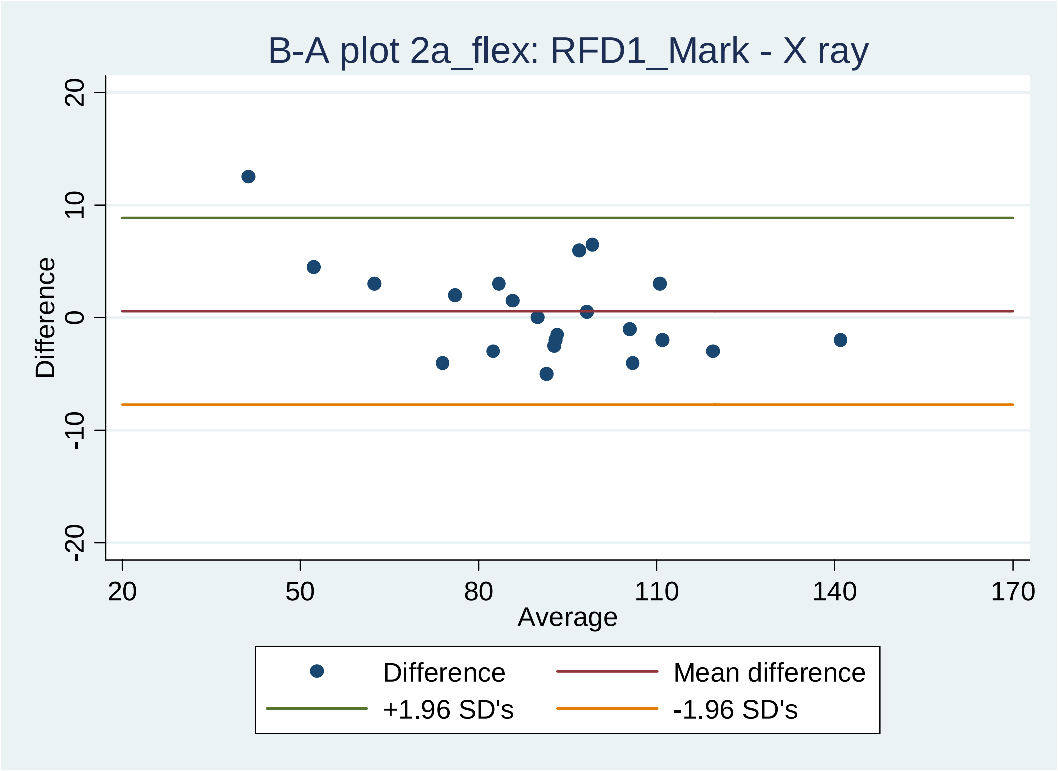
B-A plot 2a_flex: RFD1_Mark - X ray
Difference
20
10
0
-10
-20
20
50
80
110
140
170
Average
Difference
Mean difference
+1.96 SD's
-1.96 SD's

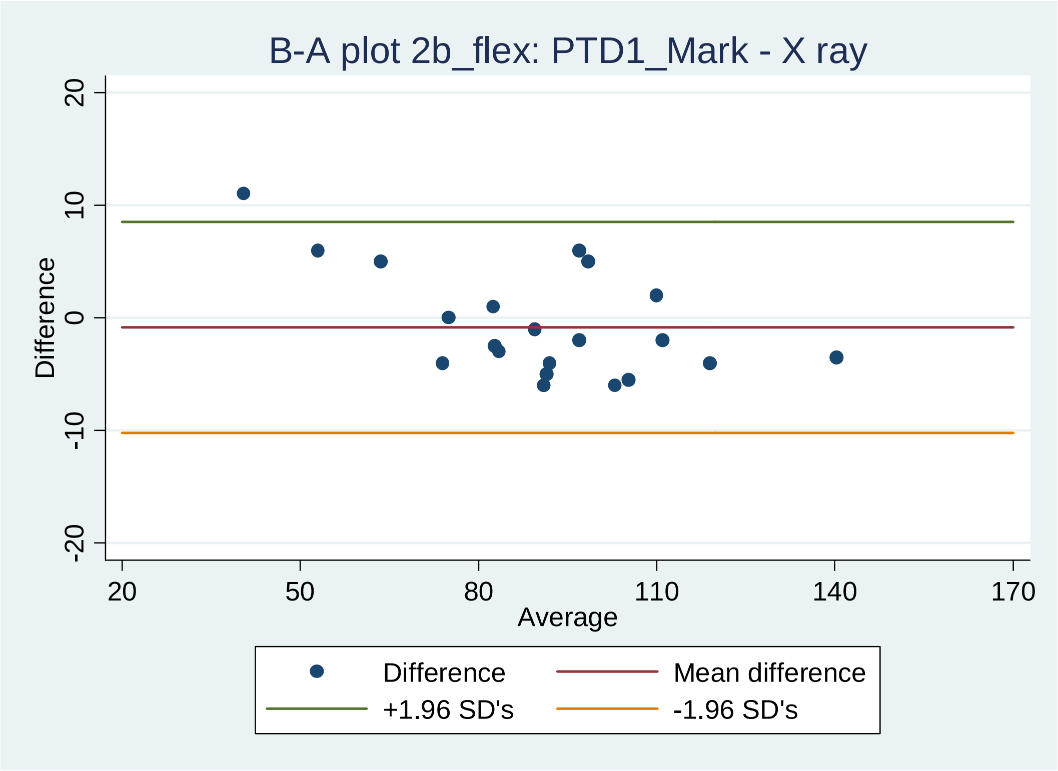
B-A plot 2b_flex: PTD1_Mark - X ray
Difference
20
10
0
-10
-20
20
50
80
110
140
170
Average
Difference
Mean difference
+1.96 SD's
-1.96 SD's

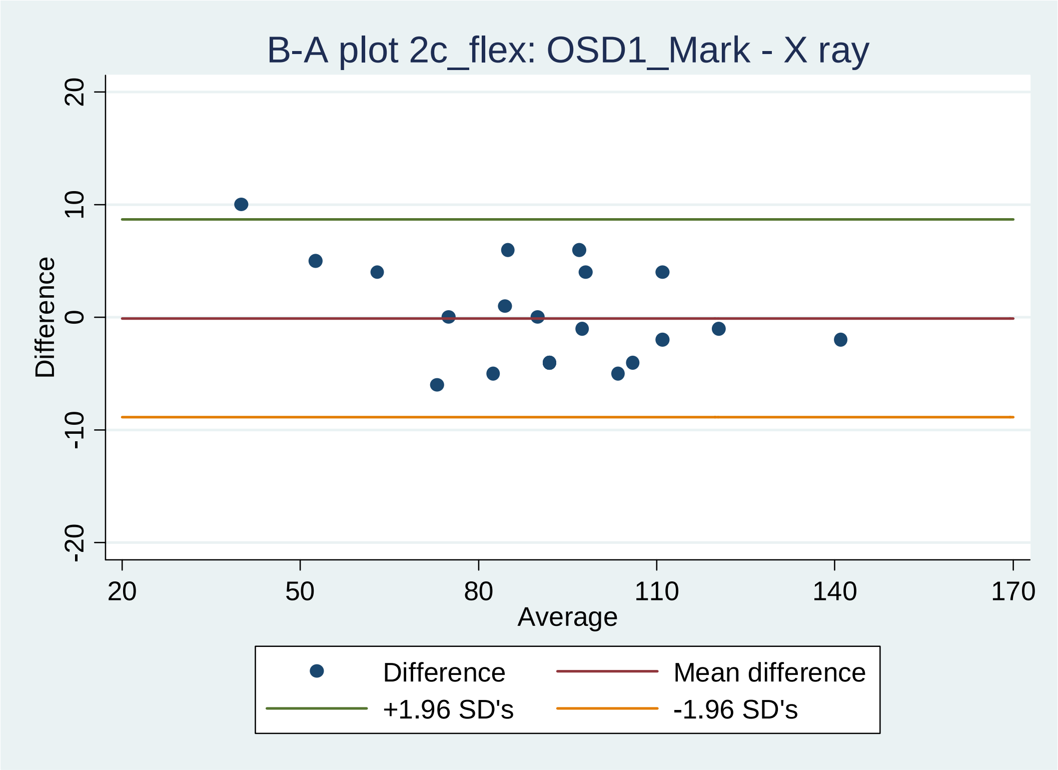
B-A plot 2c_flex: OSD1_Mark - X ray
Difference
20
10
0
-10
-20
20
50
80
110
140
170
Average
Difference
Mean difference
+1.96 SD's
-1.96 SD's

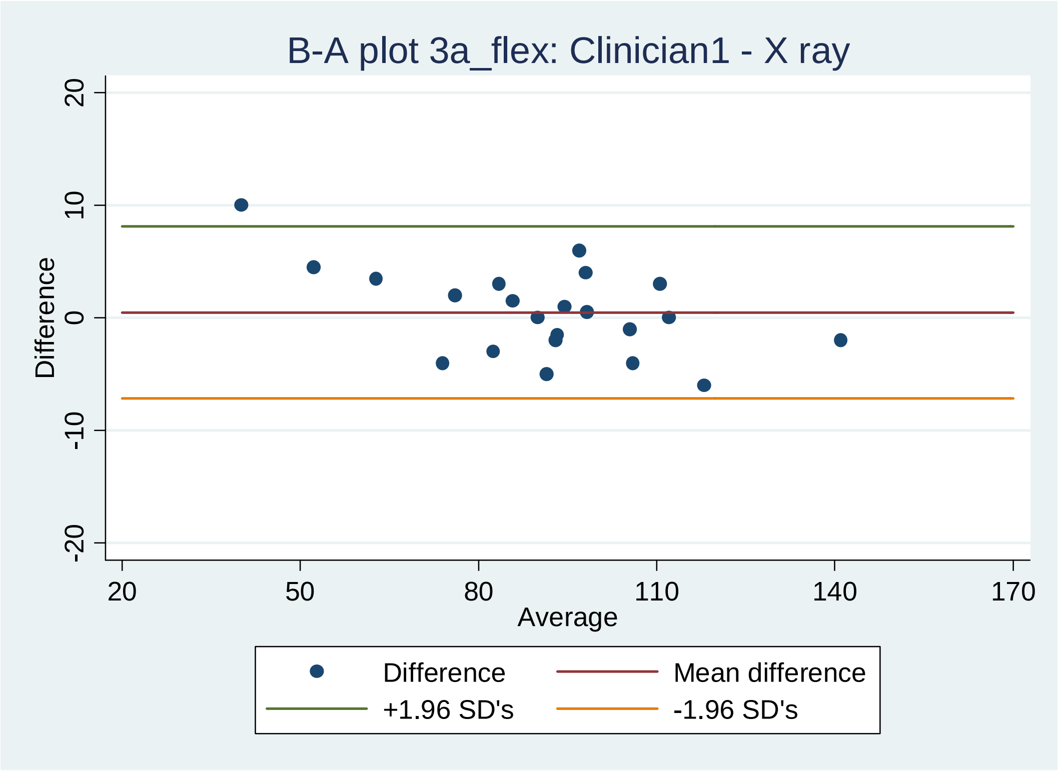
B-A plot 3a_flex: Clinician1 - X ray
Difference
20
10
0
-10
-20
20
50
80
110
140
170
Average
Difference
Mean difference
+1.96 SD's
-1.96 SD's

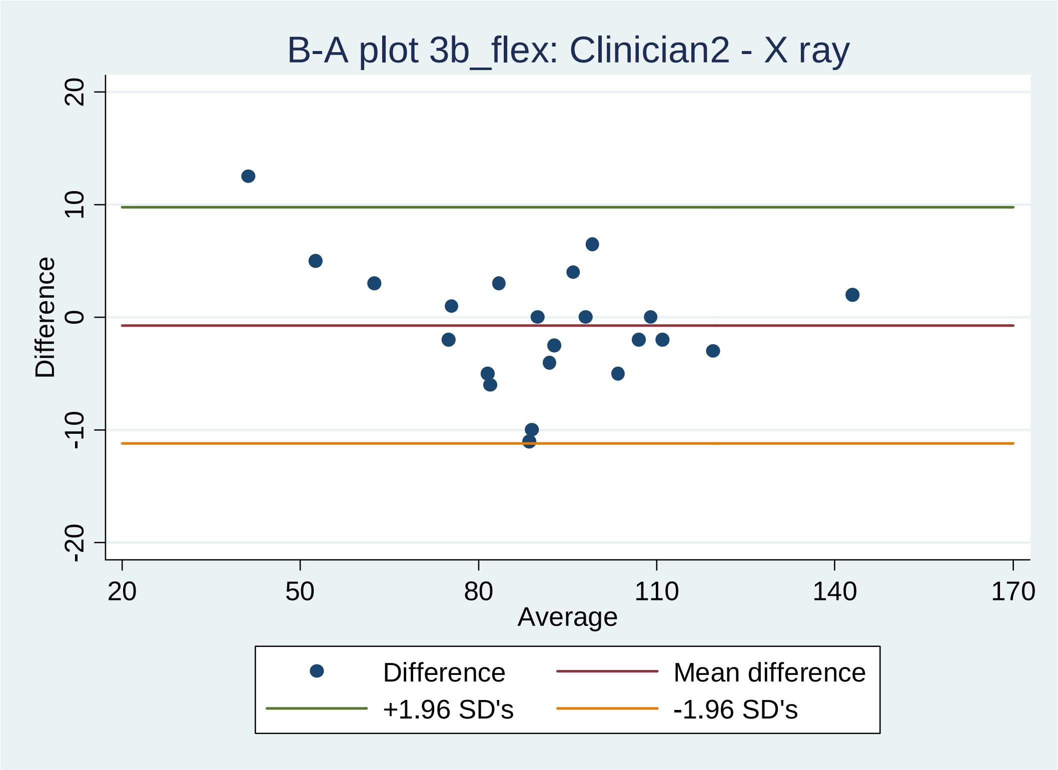
B-A plot 3b_flex: Clinician2 - X ray
Difference
20
10
0
-10
-20
20
50
80
110
140
170
Average
Difference
Mean difference
+1.96 SD's
-1.96 SD's

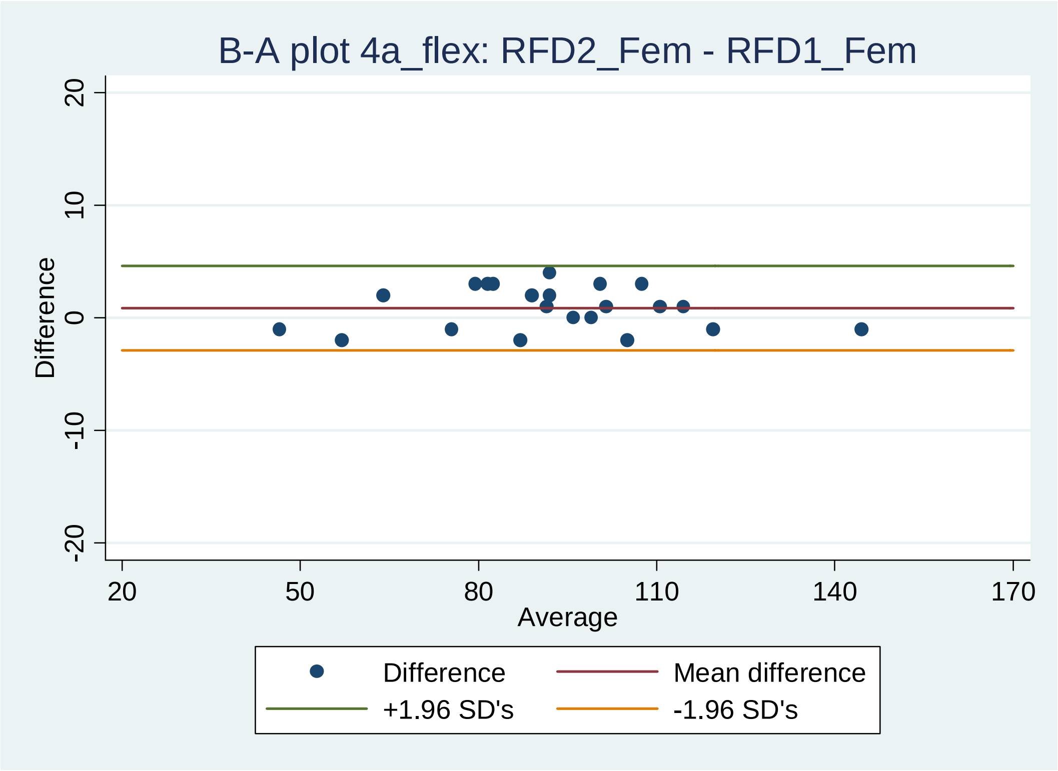
B-A plot 4a_flex: RFD2_Fem - RFD1_Fem
Difference
20
10
0
-10
-20
20
50
80
110
140
170
Average
Difference
Mean difference
+1.96 SD's
-1.96 SD's

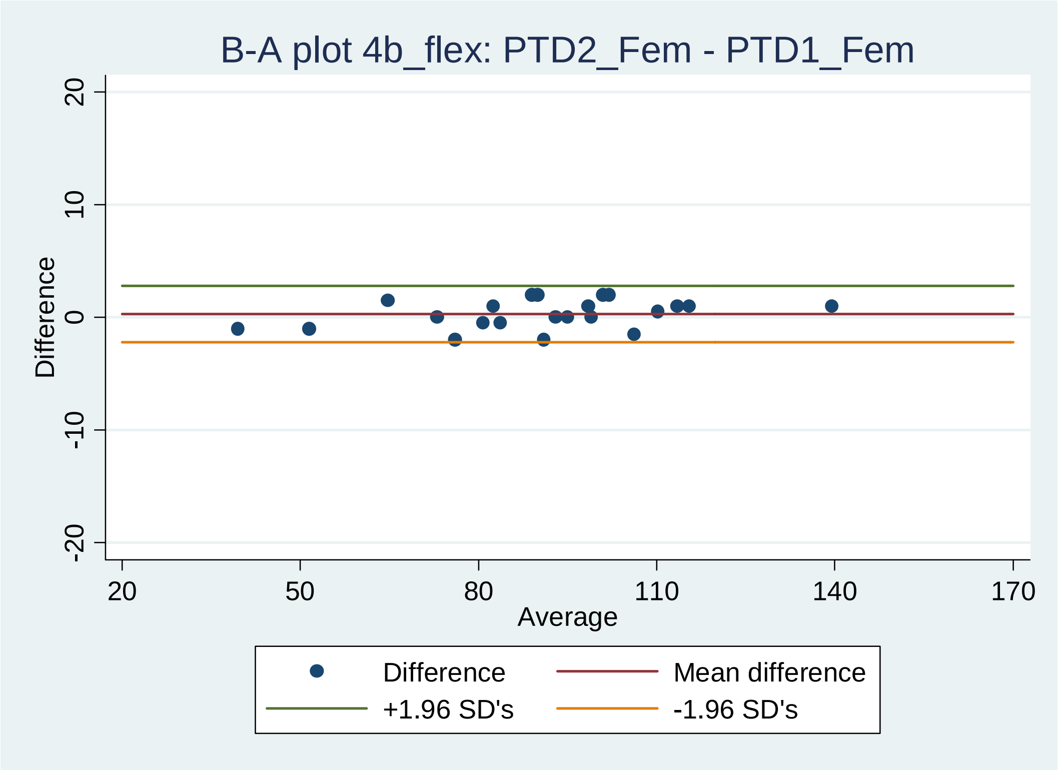
B-A plot 4b_flex: PTD2_Fem - PTD1_Fem
Difference
20
10
0
-10
-20
20
50
80
110
140
170
Average
Difference
Mean difference
+1.96 SD's
-1.96 SD's

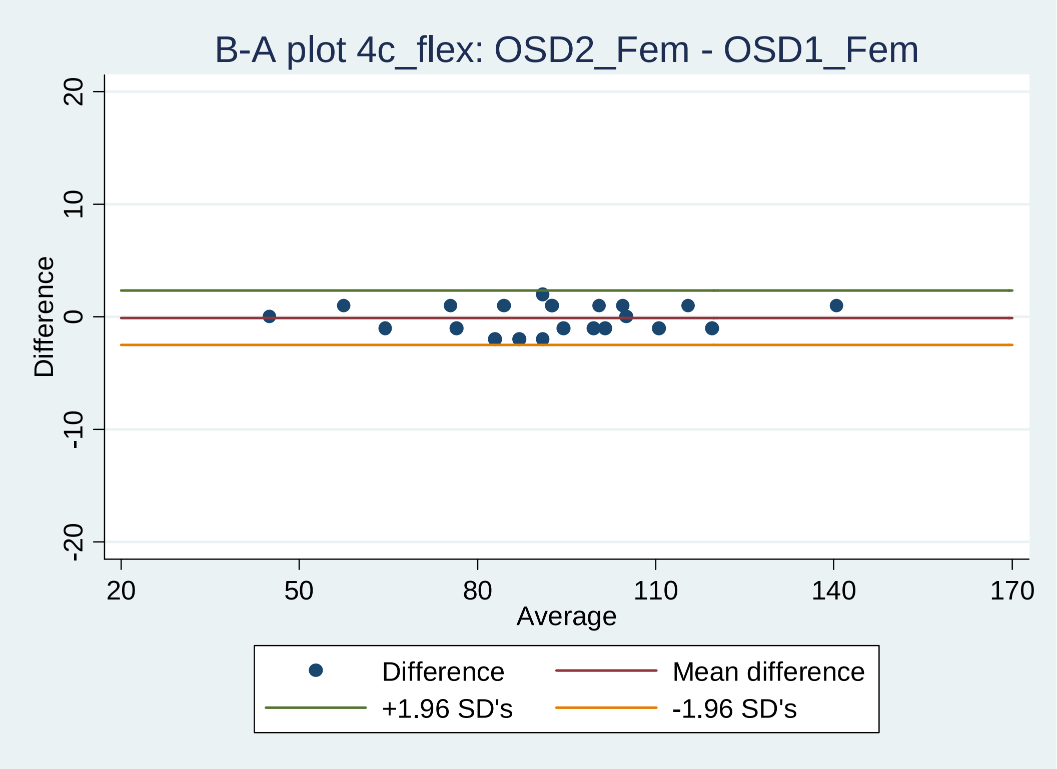
B-A plot 4c_flex: OSD2_Fem - OSD1_Fem
Difference
20
10
0
-10
-20
20
50
80
110
140
170
Average
Difference
Mean difference
+1.96 SD's
-1.96 SD's

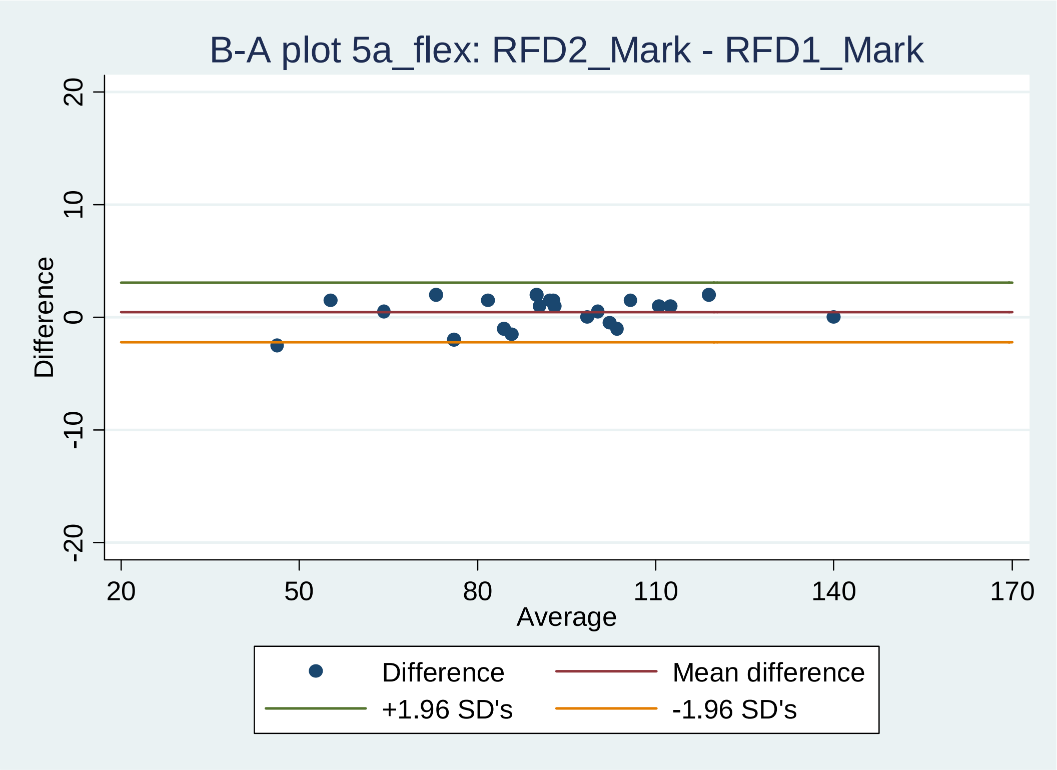
B-A plot 5a_flex: RFD2_Mark - RFD1_Mark
Difference
20
10
0
-10
-20
20
50
80
110
140
170
Average
Difference
Mean difference
+1.96 SD's
-1.96 SD's

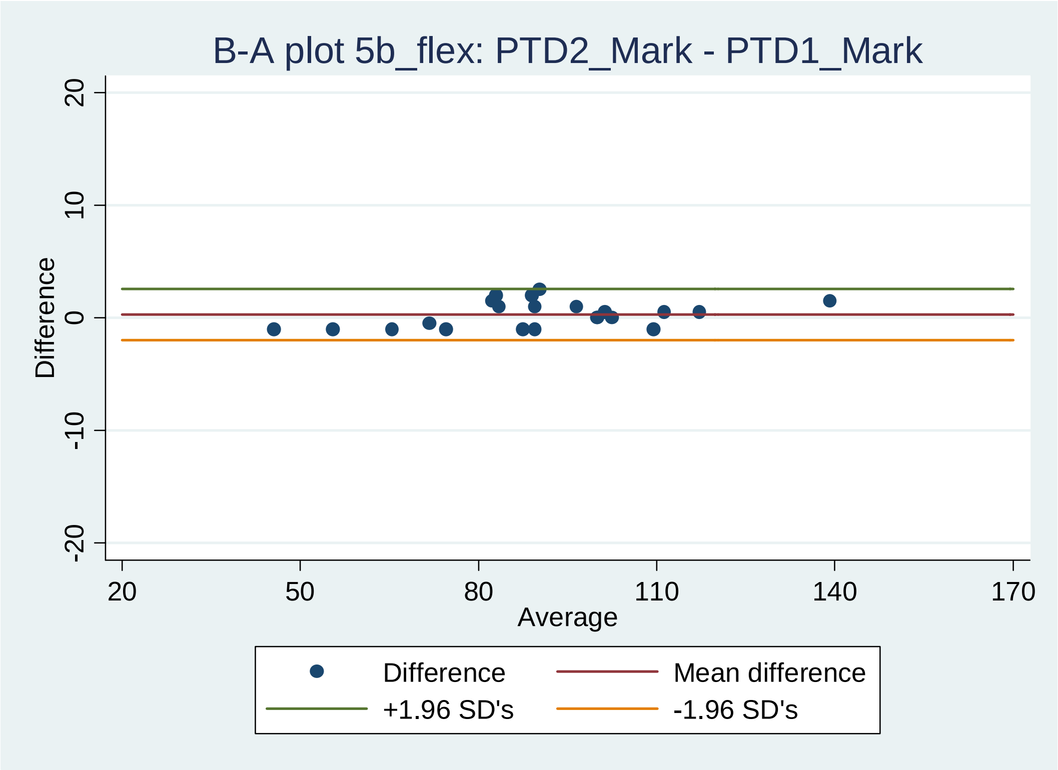
B-A plot 5b_flex: PTD2_Mark - PTD1_Mark
Difference
20
10
0
-10
-20
20
50
80
110
140
170
Average
Difference
Mean difference
+1.96 SD's
-1.96 SD's

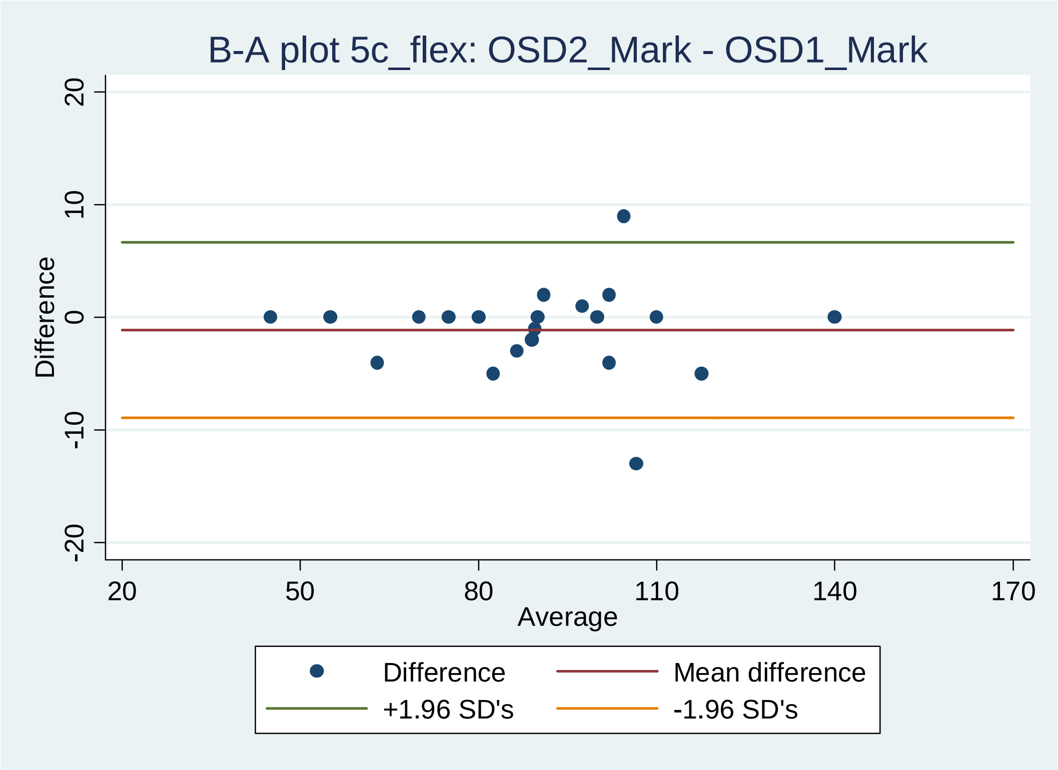
B-A plot 5c_flex: OSD2_Mark - OSD1_Mark
Difference
20
10
0
-10
-20
20
50
80
110
140
170
Average
Difference
Mean difference
+1.96 SD's
-1.96 SD's

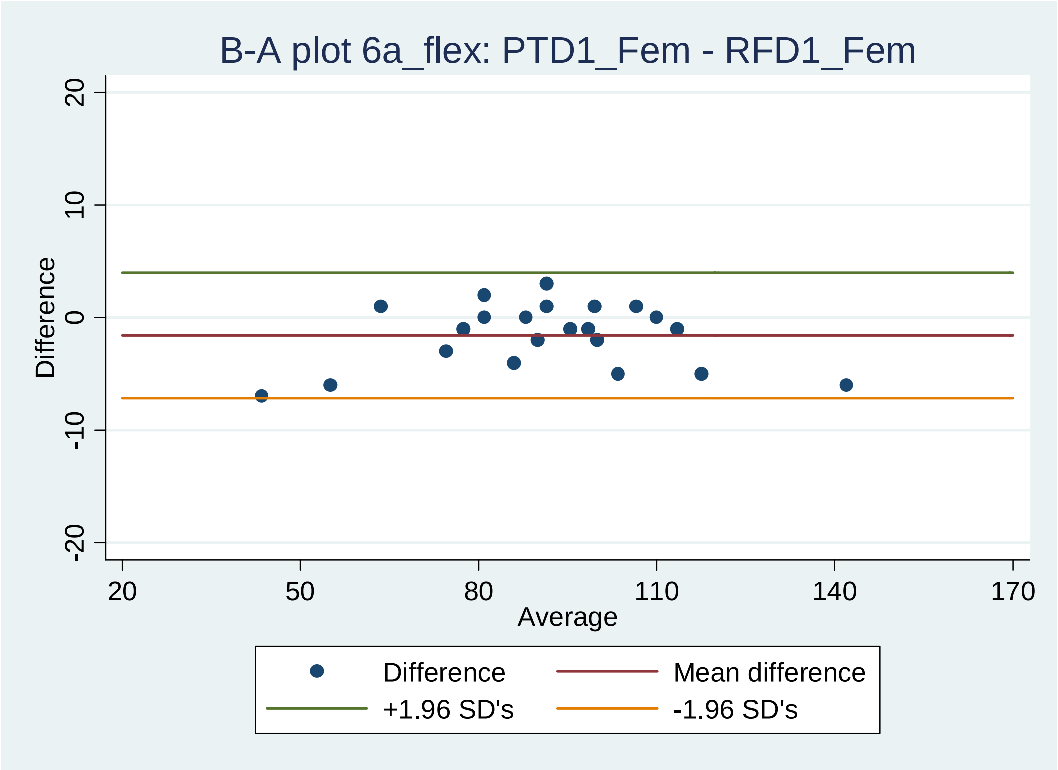
B-A plot 6a_flex: PTD1_Fem - RFD1_Fem
Difference
20
10
0
-10
-20
20
50
80
110
140
170
Average
Difference
Mean difference
+1.96 SD's
-1.96 SD's

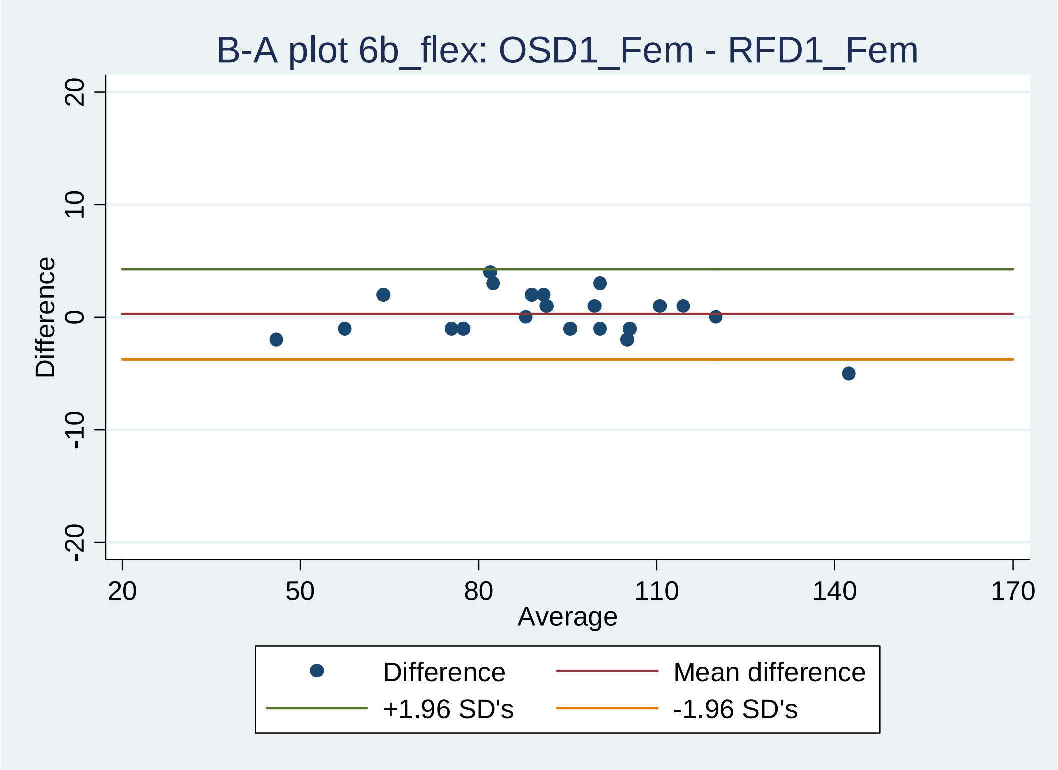
B-A plot 6b_flex: OSD1_Fem - RFD1_Fem
Difference
20
10
0
-10
-20
20
50
80
110
140
170
Average
Difference
Mean difference
+1.96 SD's
-1.96 SD's

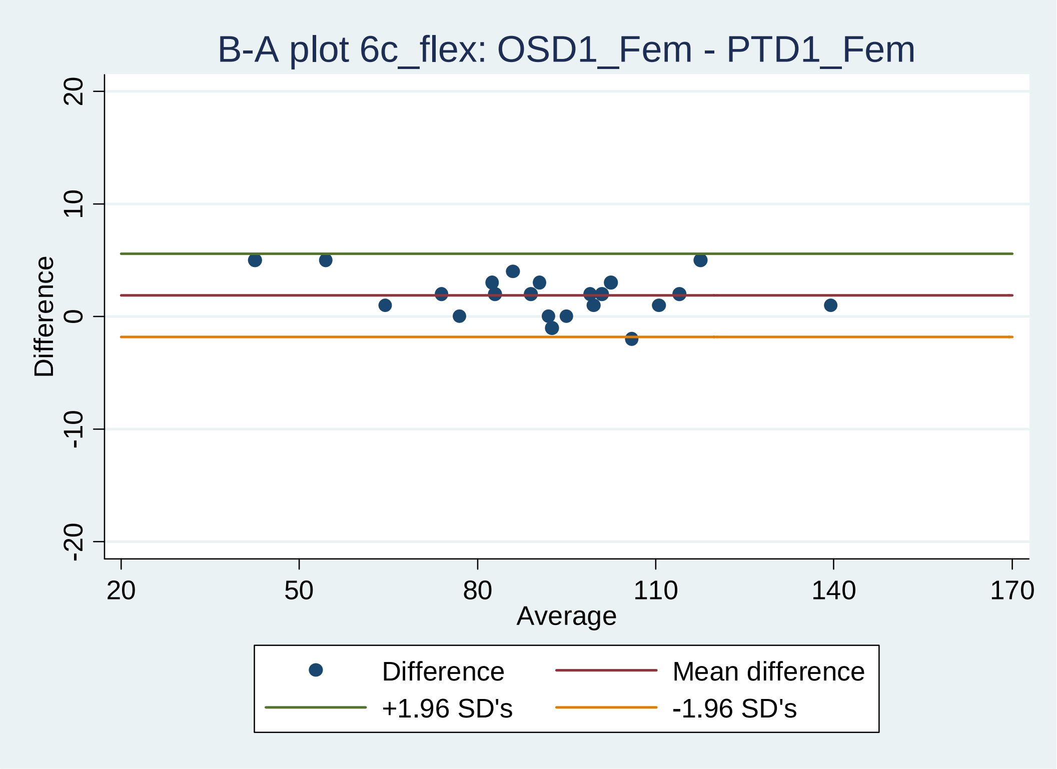
B-A plot 6c_flex: OSD1_Fem - PTD1_Fem
Difference
-20
-10
0
10
20
20
50
80
110
140
170
Average
Difference
Mean difference
+1.96 SD's
-1.96 SD's

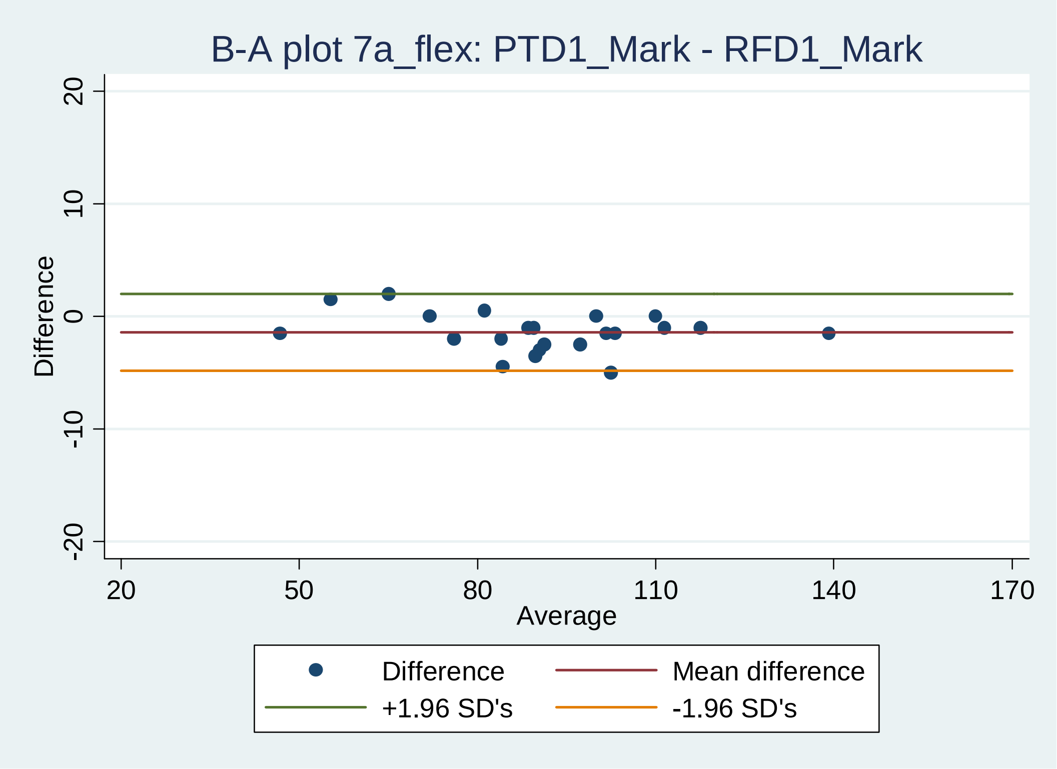
B-A plot 7a_flex: PTD1_Mark - RFD1_Mark
Difference
-20
-10
0
10
20
20
50
80
110
140
170
Average
Difference
Mean difference
+1.96 SD's
-1.96 SD's

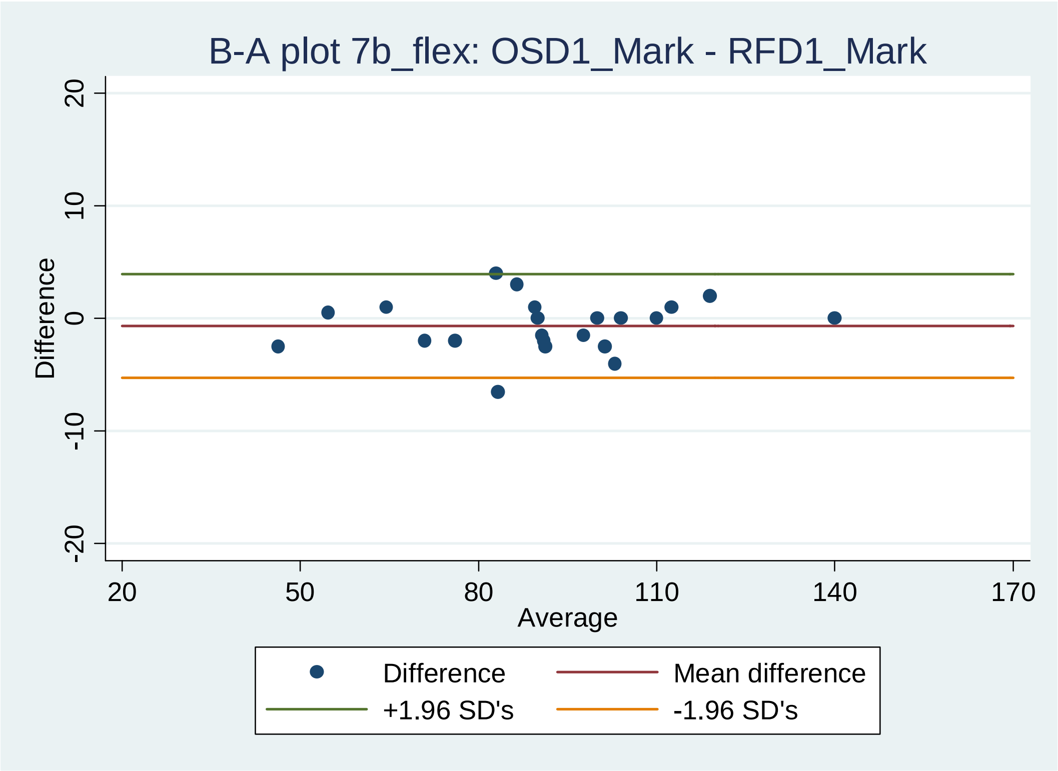
B-A plot 7b_flex: OSD1_Mark - RFD1_Mark
Difference
20
10
0
-10
-20
20
50
80
110
140
170
Average
Difference
Mean difference
+1.96 SD's
-1.96 SD's

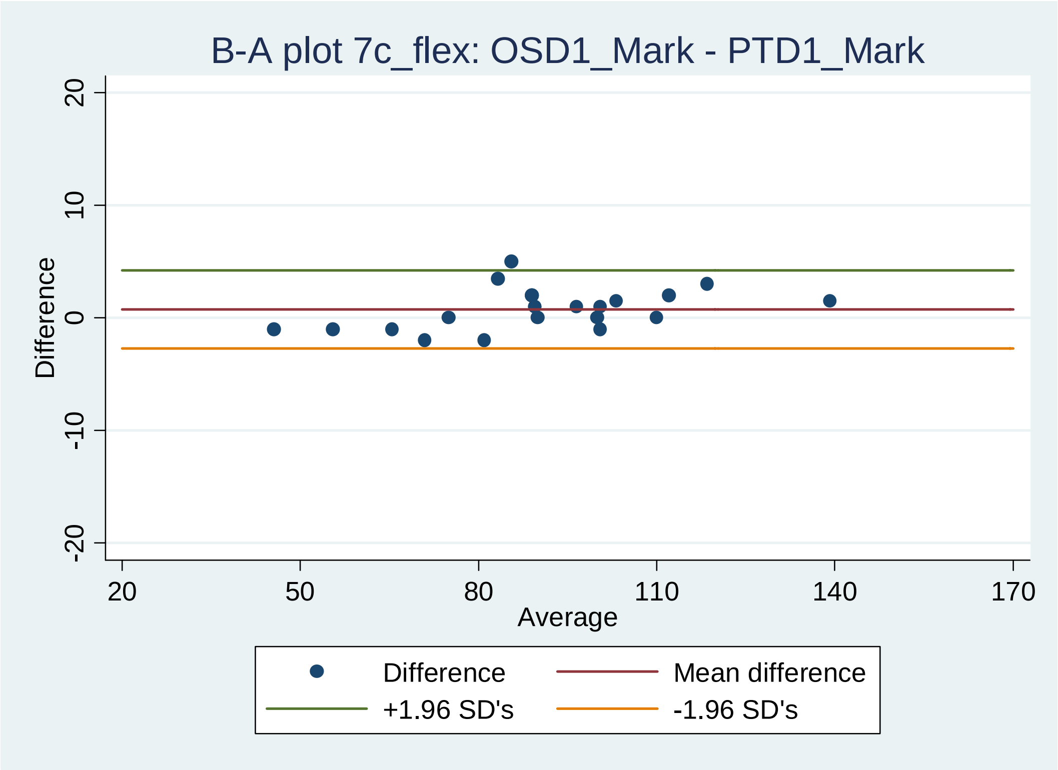
B-A plot 7c_flex: OSD1_Mark - PTD1_Mark
Difference
-20
-10
0
10
20
Average
20
50
80
110
140
170
Difference
Mean difference
+1.96 SD's
-1.96 SD's

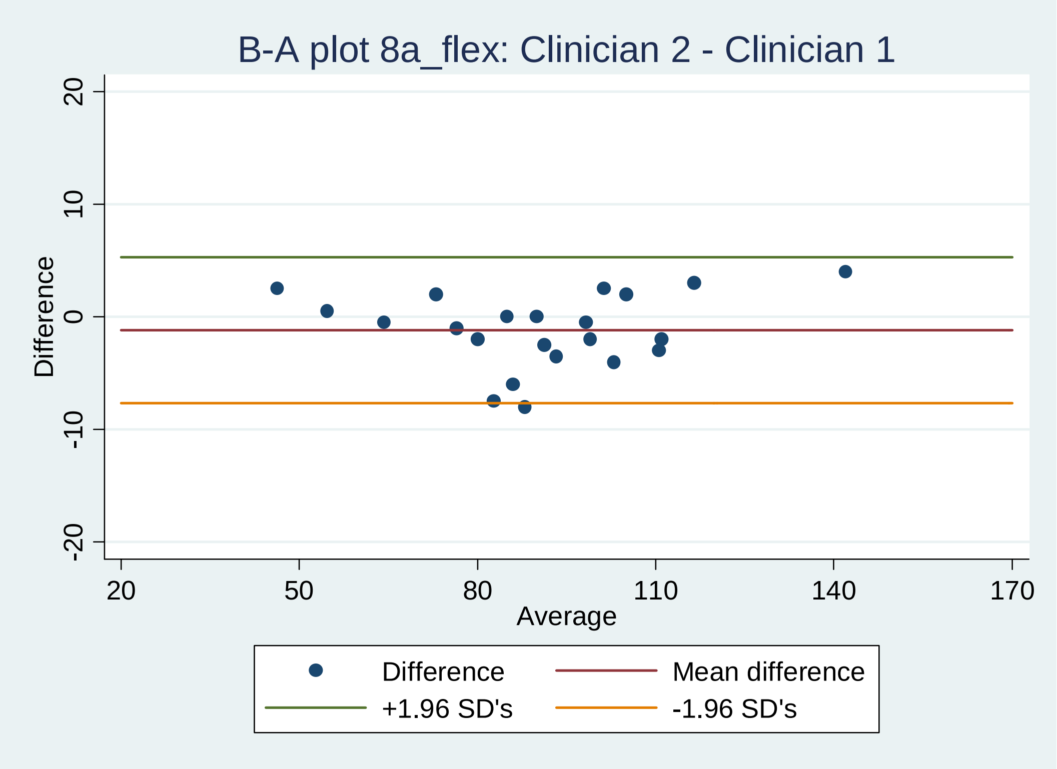
B-A plot 8a_flex: Clinician 2 - Clinician 1
Difference
-20
-10
0
10
20
20
50
80
110
140
170
Average
Difference
Mean difference
+1.96 SD's
-1.96 SD's
